# A family of diastereomeric dodecanuclear coordination cages based on inversion of chirality of individual triangular cyclic helicate faces†

**DOI:** 10.1039/d0sc04347h

**Published:** 2020-09-08

**Authors:** Stephen P. Argent, Fiona C. Jackson, Ho Man Chan, Sam Meyrick, Christopher G. P. Taylor, Tanya K. Ronson, Jonathan P. Rourke, Michael D. Ward

**Affiliations:** Department of Chemistry, University of Warwick Coventry CV4 7AL UK m.d.ward@warwick.ac.uk; School of Chemistry, University of Nottingham University Park Nottingham NG7 2RD UK stephen.argent@nottingham.ac.uk; University Chemistry Laboratory, University of Cambridge Lensfield Road Cambridge CB2 1EW UK; School of Chemistry, Cardiff University Main Building, Park Place Cardiff CF10 3AT UK

## Abstract

The dodecanuclear coordination cage [Cd_12_(L^naph^)_12_(L^mes^)_4_](BF_4_)_24_ consists of a set of four triangular, trinuclear helical panels {Cd_3_(μ-L^naph^)_3_}^6+^ (based on ditopic bridging ligands L^naph^), which are connected by four tritopic ligands L^mes^. The result is that the four triangular helical panels and the four L^mes^-capped triangular faces of the cuboctahedral core form two alternating subsets of the eight triangular faces of the cuboctahedron. Crystallographic investigations revealed that the triangular helicate faces can have ‘clockwise’ (C) or ‘anticlockwise’ (A) helicity, and that the helicity of each face can vary independently of the others as they are mechanically separated. This generates a set of three diastereoisomers in which all four cyclic helicate faces in the cuboctahedron have the same chirality (AAAA/CCCC enantiomers with *T* symmetry; AAAC/CCCA enantiomers with *C*_3_ symmetry; and achiral AACC with *S*_4_ symmetry). This mirrors the known behaviour of many simpler M_4_L_6_ tetrahedral cages which can likewise exist as *T*, *C*_3_ or *S*_4_ isomers according to the sense of tris-chelate chirality around each individual metal centre: but here it is translated onto a much larger scale by the four chiral units being entire trinuclear helicate faces rather than single metal centres. ^1^H NMR spectroscopy confirms the presence of the three diastereoisomers with their different molecular symmetries in a ratio slightly different from what is expected on purely statistical grounds; and ^1^H NMR measurements on a non-equilibrium sample (enriched by manual crystal-picking before preparing the solution) showed that the distribution does not change over several weeks in solution, indicating the kinetic inertness of the cage assemblies.

## Abstract 2

All three diastereoisomers of a cuboctahedral coordination cage containing square and triangular faces, based on different chirality of each triangular face independently, have been structurally characterised.

## Graphical abstract

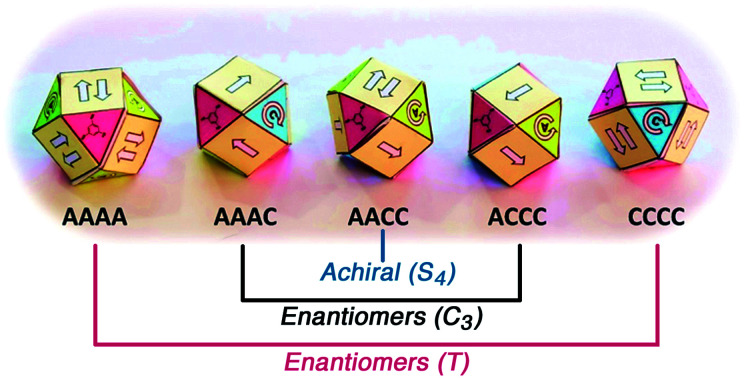

## Introduction

The assembly of combinations of metal ions and multi-topic bridging ligands into coordination cages is now a mature field which has afforded a huge range of beautiful species of interest for their structural diversity^1–5^ and the functions arising from their host–guest chemistry.^2^ A large subset of these, based on square planar Pd(ii)/Pt(ii) and rigid linear or panel-like planar ligands,^3^ illustrate how elaborate structures can be assembled with a high degree of predictability once the underlying structural and geometric principles are appreciated. In contrast, the use of octahedral tris-chelate vertices provides two additional sources of structural diversity which are less predictable. These are (i) the possibility of *fac*/*mer* isomerism at individual metal centres in a multinuclear assembly,^4^ and (ii) the chirality at each centre associated with the configuration in which the three chelate units assemble around the metal. Chirality in large self-assembled systems is a highly important and topical field.^5^

An interesting consequence of incorporation of multiple octahedral tris-chelate metal centres into cages is the possibility of diastereoisomerism arising from inversion of configuration at one centre but not others, affording a family of related but structurally distinct complexes. This is best illustrated by some examples of M_4_L_6_ tetrahedral complexes in which bis-bidentate bridging ligands span the cage edges. Usually such tetrahedral cages form homochiral assemblies (as racemates), *i.e.* they have ΛΛΛΛ/ΔΔΔΔ configurations with *T* molecular symmetry.^6^ However they can also sometimes form with one vertex inverted with respect to the other three, *i.e.* they have ΛΛΛΔ/ΔΔΔΛ configurations with *C*_3_ molecular symmetry; or two vertices can be inverted giving achiral ΛΛΔΔ assemblies with *S*_4_ molecular symmetry. A few such systems where all three diastereoisomers are present in equilibrium have been reported.^7^

Here, we report a novel extension of this principle in the form of a diastereomeric family of dodecanuclear cuboctahedral coordination cages in which the structural variation is provided not by inversion of individual metal vertices, but by inversion of chirality of entire triangular cyclic helicate faces. Inverting the chirality of entire triangular panels, independently of one another, in a polyhedral assembly provides an intriguing addition to the structural diversity of these cages. Using purely organic assemblies and covalent bond formation, Cao and co-workers have demonstrated how different enantiomers of a three-fold symmetric truxene unit could be incorporated into the triangular faces of an octahedral assembly, with different diastereoisomers of the cage being formed according to the disposition of truxene units of different chirality around the faces.^8^ The examples we report here, based on self-assembly using achiral ligands and labile metal ions, constitute the first such example in the field of metal-directed assembly of coordination cages. All three diastereomeric forms of the cuboctahedral cage have been identified and structurally characterised, with ^1^H and ^113^Cd NMR spectroscopy confirming the existence of the mixture of diastereomers in solution.

## Results and discussion

**Scheme 1:**
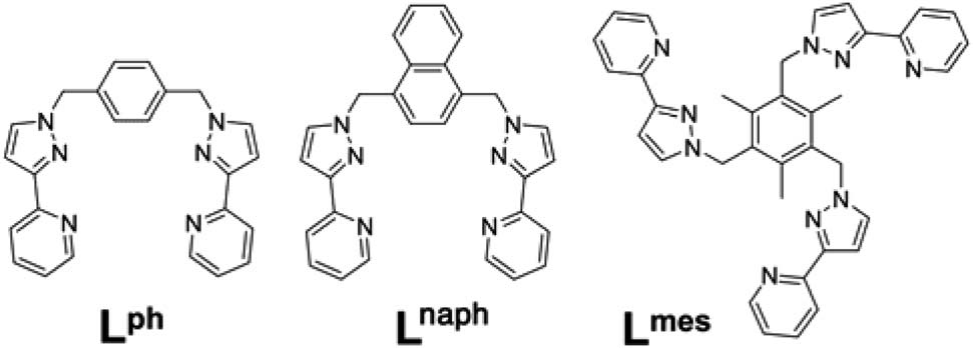
Structural formulae of the face-capping ligand L^mes^ and the edge-bridging ligands L^ph^ and L^naph^ referred to in this paper.

**Fig. 1:**
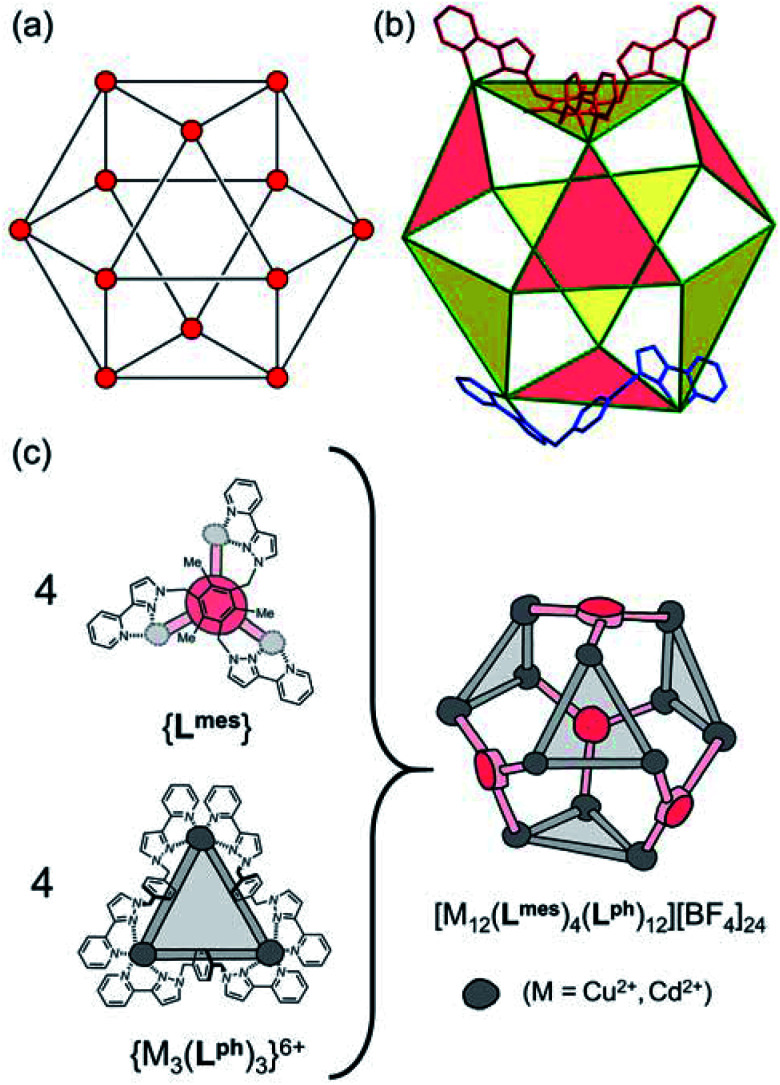
(a) An idealised cuboctahedron; (b) partial crystal structure of [Cu_12_(L^ph^)_12_(L^mes^)_4_](BF_4_)_24_ (taken from ref. 9*b*) emphasising the cuboctahedral Cu_12_ core with the four triangular faces capped by tritopic ligands L^mes^ coloured yellow, and the alternate four triangular faces – which are Cu_3_(μ-L^ph^)_3_ cyclic helicates – coloured pink; (c) additional sketch emphasising the disposition of the four M_3_(μ-L^ph^)_3_ cyclic helicate faces in these structures (grey triangles) and the four L^mes^ ligands which connect them (red).

Several years ago we reported the assembly and crystal structures of some unusual cuboctahedral cage complexes that formed by selection of two different ligands from a mixture during the assembly process (Scheme 1 and Fig. 1).^9^

The complexes [M_12_(L^ph^)_12_(L^mes^)_4_]X_24_ (M = Cu, Cd; X = ClO_4_ or BF_4_) contain a cuboctahedral core of twelve M(ii) ions which contains eight triangular faces and six square faces. The cuboctahedral structure may be considered as derived from a cube, with the eight corners truncated to reveal triangular faces whose vertices meet exactly in the centre of what used to be an edge of the cube. With all vertices equivalent but with two different types of face, the cuboctahedron is one of the class of Archimedean solids. The two types of ligand (ditopic L^ph^ is edge-bridging and tritopic L^mes^ is face-capping) are associated with different subsets of the eight triangular faces; these eight faces can thus be split into two subsets of four (Fig. 1b), which alternate around the cage and each describe a tetrahedron. In one subset of four triangular faces, each face is capped by a tripodal L^mes^ ligand. In the other subset, each of the four triangular faces contains a set of three L^ph^ ligands around the edges, with all ligands having a helical twist such that each triangular face is a M_3_(μ-L^ph^)_3_ cyclic helicate.^9^ The assembly can be regarded as a tetrahedral array of four M_3_(μ-L^ph^)_3_ cyclic helicates, each with a vacant site for an additional chelating ligand at each metal ion, connected by four L^mes^ ligands: this way of considering the structure is emphasised in Fig. 1c.

For the Cd_12_ cage of this type that we reported earlier,^9^ we observed three ^113^Cd environments by NMR, implying a loss of regular cuboctahedral symmetry in which all metal ions would be equivalent. This was unexpected and a consequence of the fact that two of the four triangular cyclic helical faces have ‘clockwise’ helicity (which we denote ‘C’) and the other two have ‘anticlockwise’ helicity (which we denote ‘A’) giving a CCAA combination of face chiralities. This results in (non-crystallographic) *S*_4_ molecular symmetry such that one quarter of each cage – *i.e.* a subset of three metal ions and the associated ligands – is unique.^9,10^ As this structural type was the only product isolated for each of the original [M_12_(L^ph^)_12_(L^mes^)_4_]X_24_ cages^9^ no particular significance was attached to this at the time, but it becomes relevant to this new work.

Motivated by the ability of some members of our cage family to act as excellent hosts for small molecule guests,^11^ we have revisited this cuboctahedral cage structure with the intention of investigating its host/guest chemistry. We also wished to incorporate fluorophores into the ligand array which might act as luminescent reporters of guest binding, or which might perform photoinduced electron transfer or energy transfer to bound guests in the manner that we have recently demonstrated with different photo-active cages.^12^ Accordingly we used the same methodology as was used for preparation of the original cuboctahedral complexes [M_12_(L^ph^)_12_(L^mes^)_4_]X_24_,^9^ but have replaced the edge-bridging ligand L^ph^ with L^naph^ (Scheme 1)^13^ to incorporate the fluorescent groups that we wanted. Examination of the structures of the original cages [M_12_(L^ph^)_12_(L^mes^)_4_]X_24_ indicated that incorporation of the additional aromatic rings by replacing L^ph^ with L^naph^ should not present a significant steric barrier to cage assembly, and so it proved.

**Fig. 2:**
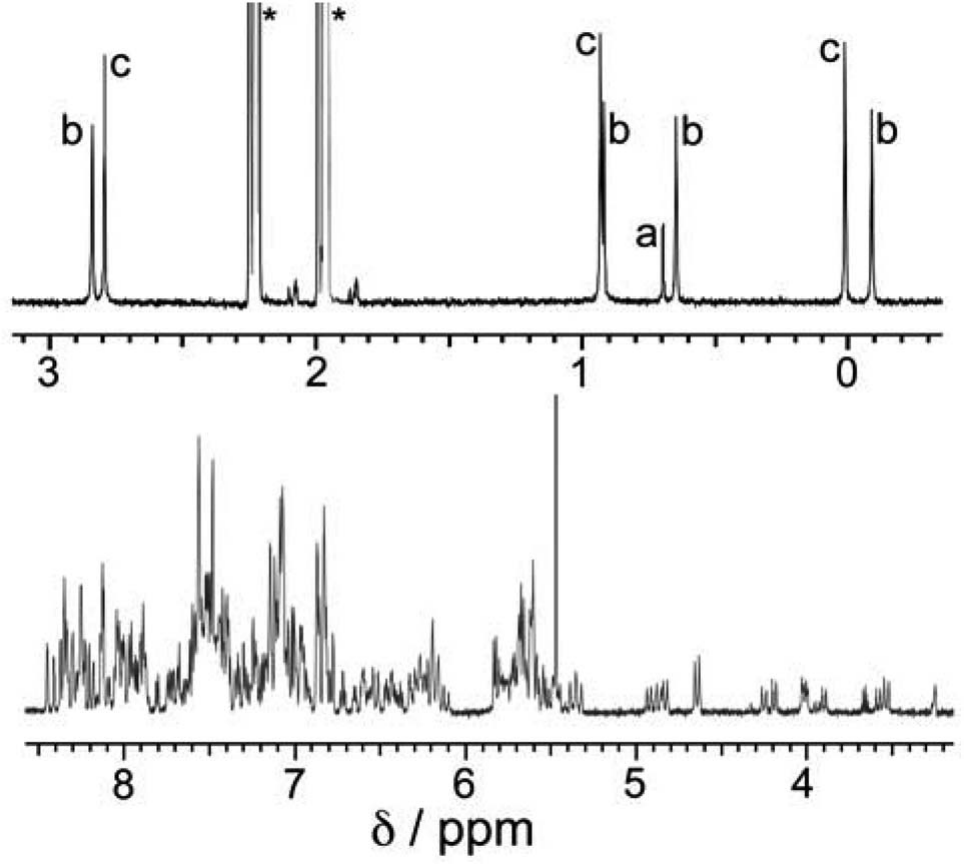
600 MHz ^1^H NMR spectrum of a sample of as-prepared [Cd_12_(L^naph^)_12_(L^mes^)_4_](BF_4_)_24_ (mixture of isomers) in CD_3_CN. The aliphatic region (0–3 ppm) is shown on an expanded scale to clarify the sets of signals associated with the L^mes^ methyl groups: these are labelled ‘a’ (one signal for the *T* isomer), ‘b’ (four signals of equal intensity for the *C*_3_ isomer) and ‘c’ (three signals of equal intensity for the *S*_4_ isomer). The relative intensities of signals in each group a/b/c are 1 : 2.4 : 3.2, indicating a composition of 5% (*T*) : 47.5% (*C*_3_) : 47.5% (*S*_4_) in the mixture – a deficiency of the *T* isomer and an excess of the *S*_4_ isomer compared to expectations based on a purely statistical distribution [12.5% (*T*) : 50% (*C*_3_) : 37.5% (*S*_4_) – see main text and footnote].

Reaction of L^naph^, L^mes^ and Cd(BF_4_)_2_ in a 3 : 1 : 3 ratio, as required for assembly of the desired cuboctahedral cage, in acetonitrile followed by rapid precipitation upon addition of diisopropyl ether afforded a colourless crystalline solid which proved to the desired product [Cd_12_(L^naph^)_12_(L^mes^)_4_](BF_4_)_24_. ES mass spectrometry confirmed the formulation, showing a characteristic sequence of peaks corresponding to {[Cd_12_(L^naph^)_12_(L^mes^)_4_](BF_4_)_24−*n*_}^*n*+^ (*n* = 4–9), *i.e.* the intact cage cation associated with varying numbers of anions (Fig. S1†). ^1^H NMR spectroscopy (Fig. 2) revealed a very complex spectrum with numerous sub-spectra of different intensities, clearly indicating the presence of a mixture of species. This is particularly clearly apparent in the 0–3 ppm region where the signals for the methyl groups of L^mes^ appear; these provide a much more convenient diagnostic handle than the aromatic region where numerous signals overlap.

Recrystallisation of the material by slow diffusion of di(isopropyl)ether vapour into a solution of the material in MeCN afforded single crystals with different habits: unit cell determination on many of these revealed that three different unit cells could be identified consistently, all occurring as a mixture in each batch of crystals. Accordingly we collected all three data sets and found on solving and refining the structures that we have isolated three different diastereoisomers of the expected cuboctahedral cage [Cd_12_(L^naph^)_12_(L^mes^)_4_](BF_4_)_24_, with the differences arising from the helicity of the individual Cd_3_(μ^2^-L^naph^)_3_ helicate faces. We will give a general overview of this first to illustrate the principle before discussing the individual structures in detail.

The array of metal ions in a regular cuboctahedron has the same symmetry as a cube, *i.e.* point group *O*_h_, with all twelve vertices equivalent (Fig. 1). This is not affected by the presence of the L^mes^ ligands on four of the eight triangular faces, as these triangular ligands are capable – when coordinated in a face-capping mode – of preserving the threefold rotational symmetry through those faces. The isomerism arises from the fact that each of the four Cd_3_(μ-L^naph^)_3_ helicate faces can have ‘clockwise’ (C) or ‘anticlockwise’ (A) helicity, and, crucially, that these can vary independently of one another. Each Cd_3_(μ^2^-L^naph^)_3_ face (pink in Fig. 1b) is connected *via* a shared vertex to three Cd_3_(μ^3^-L^mes^) faces (yellow in Fig. 1b) in which coordination of the achiral, tripodal L^mes^ ligand is not affected by the sense of rotation of these Cd_3_(μ^2^-L^naph^)_3_ faces; each chelating arm in L^mes^ will occupy the same two coordination sites of each of its Cd(ii) ions, even if the other two chelating pyrazolyl-pyridine units (from L^naph^) have their positions changed to invert the chirality at that metal centre. Changing the helicity around one Cd_3_(μ^2^-L^naph^)_3_ face does not, therefore, trigger any ongoing requirements for major changes in ligand coordination elsewhere: the four cyclic helicate Cd_3_(μ^2^-L^naph^)_3_ faces are mechanically decoupled from one another and can act independently in this respect.

**Fig. 3:**
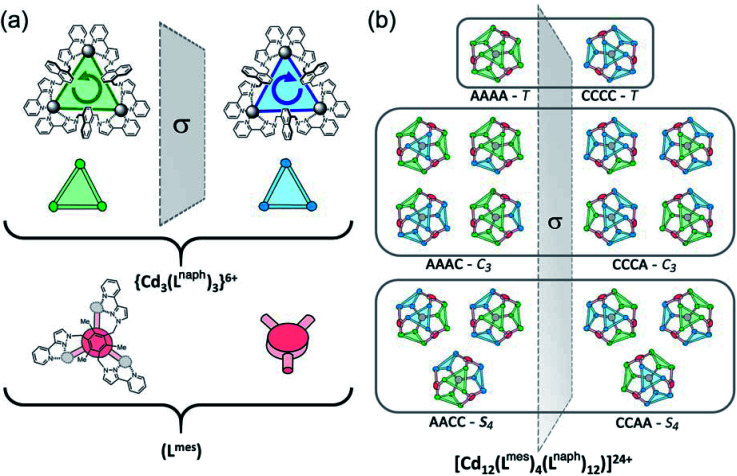
Graphical illustration of the different stereoisomers of the cage and their degeneracies: (a) illustration of the two different chiralities of the cyclic helicate triangular panels, shown as green (anticlockwise, A) and blue (clockwise, B) panels, with the connecting tripodal ligands L^mes^ shown in red; (b) illustration of the different ways in which these chiral panels can assemble into a cuboctahedral Cd_12_ array, highlighting both the different enantiomers within each diastereoisomeric structure (left and right of the central mirror plane) and the number of ways in which each diastereoisomer can be achieved (degeneracy) based on random assembly of chiral panels. Bearing in mind that the AACC and CCAA isomers in Fig. 3(b) are identical as this isomer is achiral, we can see that the five stereoisomers AAAA, AAAC, AACC, ACCC and CCCC would have a 1 : 4 : 6 : 4 : 1 binomial distribution if assembly of the A and C chiral faces were completely random. For spectroscopic purposes, given that the two enantiomers AAAA/CCCC and the two enantiomers AAAC/CCCA are equivalent by NMR spectroscopy, this would give a 2 : 8 : 6 (= 1 : 4 : 3) expected ratio of diastereoisomers, see main text.

The consequences of this are illustrated in cartoon form in Fig. 3. The two enantiomers of the cyclic helicate faces are shaded blue or green, and these are connected by the L^mes^ tripodal ligands (red). With four cyclic helicate faces arranged in a tetrahedron around the cuboctahedral core, the different combinations of helicity mirror exactly what happens in smaller M_4_L_6_ cages described above which can occur as a mixture of *T*, *C*_3_ and *S*_4_ isomers depending on whether individual metal tris-chelate centres have Λ or Δ configurations. Thus the cuboctahedron containing four chiral cyclic helicate Cd_3_(μ^2^-L^naph^)_3_ faces can in principle exist as a homochiral *T*-symmetric isomer CCCC/AAAA (which are of course enantiomers); a *C*_3_-symmetric ACCC/CAAA isomer in which one of the Cd_3_(μ^2^-L^naph^)_3_ faces has the opposite helicity to the other three; and the *S*_4_-symmetric AACC/CCAA isomer. This is shown in Fig. 3b which also emphasises the degeneracies of these structures, *i.e.* the numbers of ways in which they can form by random aggregation of the cyclic helicate faces (1 : 4 : 3 for the *T*, *C*_3_ and *S*_4_ isomers respectively).

More subtly, in these diastereomers the environments of the L^mes^ ligands will be different. In the *T* isomer these four ligands are all equivalent and all retain their threefold symmetry, such that one third of an L^mes^ ligand and one Cd atom – one twelfth of the assembly – are unique and all 12 Cd atoms are equivalent. In the *C*_3_ isomer (AAAC and its enantiomer) one L^mes^ ligand on the *C*_3_ axis is adjacent to three equivalent ‘A’ helical faces and retains its threefold symmetry; the other three L^mes^ ligands are all adjacent to two ‘A’ and one ‘C’ helical faces: they are equivalent to one another but different from the first one, and have lost their internal symmetry. This means that there are 1.333 independent L^mes^ ligands (four independent pyrazolyl-pyridine chelating arms) and hence four independent Cd atom environments (one third of the assembly). Finally in the AACC isomer all four L^mes^ ligands are equivalent, as each one is adjacent to an A/A/C (or the enantiomeric A/C/C) set of helical faces, but have no internal symmetry. Thus there is 1 unique L^mes^ ligand and hence three independent Cd atom environments (one quarter of the assembly) in this diastereomer. These considerations are important for understanding the NMR spectra which will be discussed later.

**Fig. 4:**
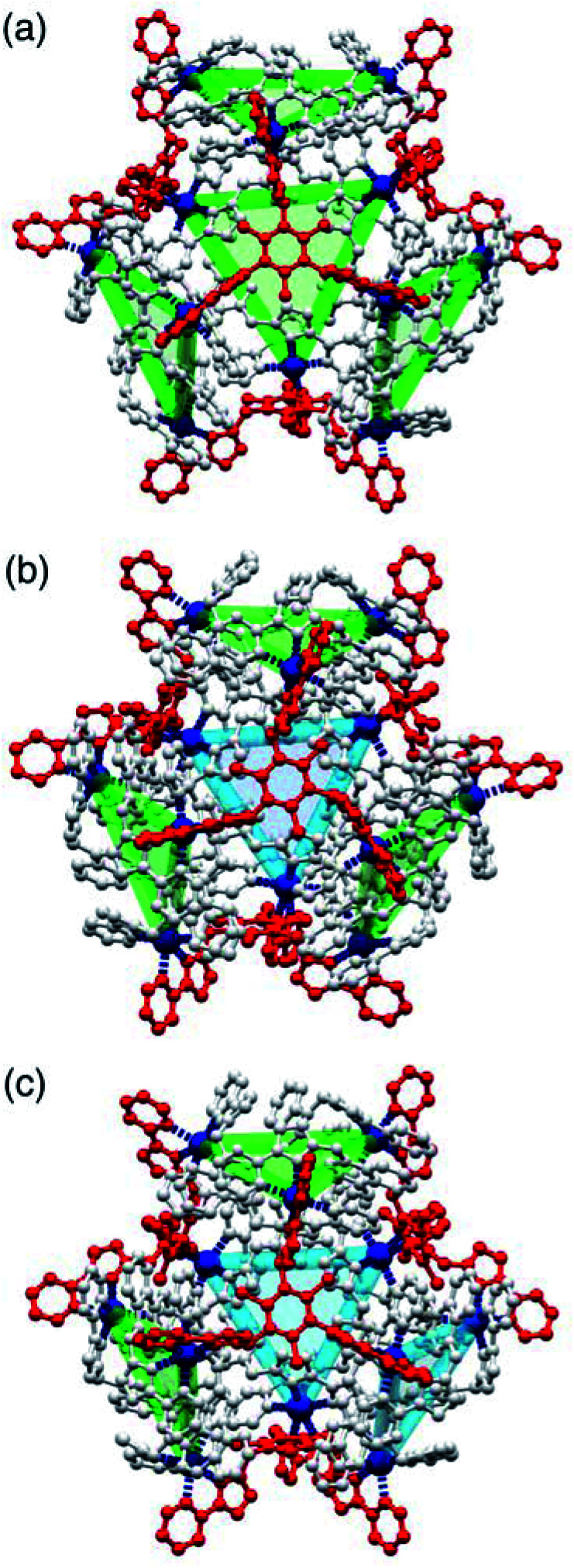
Views of the three different diastereoisomeric cuboctahedral cages [Cd_12_(L^naph^)_12_(L^mes^)_4_](BF_4_)_24_ with (a) *T*, (b) *C*_3_ and (c) *S*_4_ symmetry (these are the molecular symmetries in solution, not necessarily crystallographic). The cyclic helicate triangular panels are shown with blue and green shading, and the L^mes^ ligands are highlighted in red: the reduction in symmetry of the L^mes^ ligands down the series is clear.

**Fig. 5:**
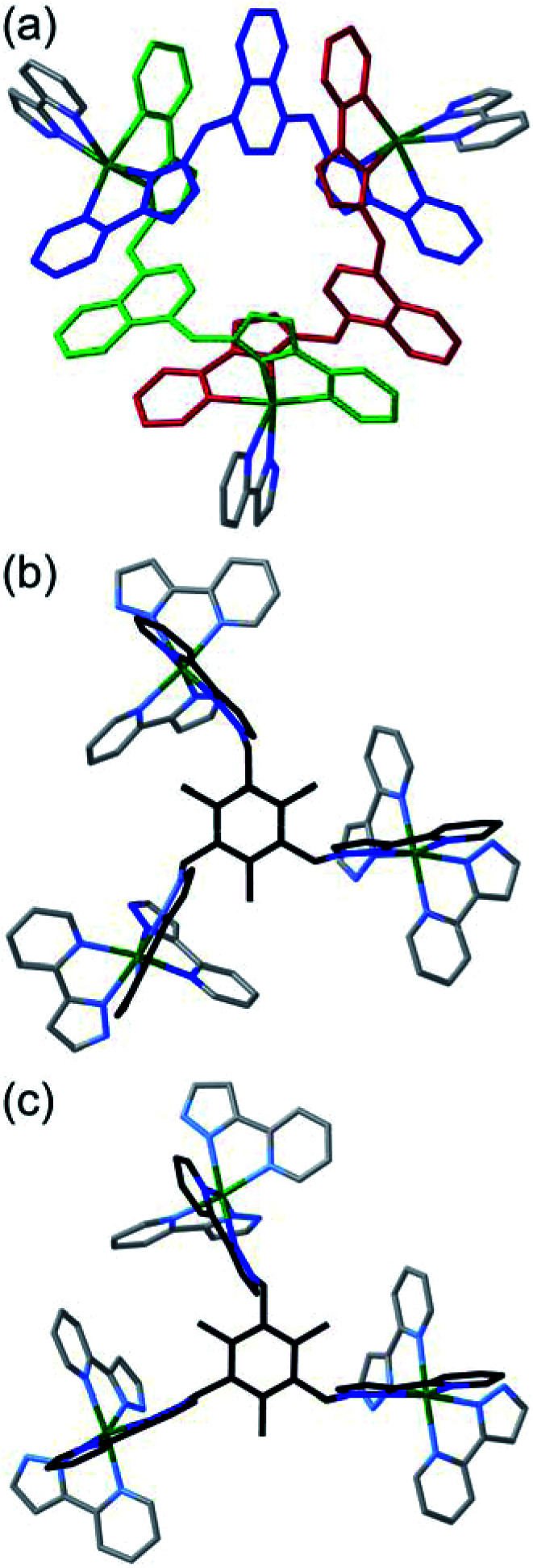
Detailed expansions of some of the structural features of the complexes. (a) A cyclic helicate face in the structure of the *T* isomer with the three (equivalent) ligands L^naph^ shown in different colours (red, green, blue) for clarity and the chelating arms of the associated L^mes^ ligands shown in grey; pi-stacking of each naphthyl group, sandwiched between two adjacent pyrazolyl-pyridine units from different ligands, is clear. Parts (b) and (c) show the two different conformations of the L^mes^ ligands in the *C*_3_ cage isomer, with (b) the unique L^mes^ ligand lying on the molecular *C*_3_ axis, and (c) one of the other three equivalent L^mes^ ligands which have no internal symmetry.

One of the crystal morphologies had the cubic space group *F*23, and this turned out to be the *T* symmetry isomer with a homochiral arrangement of the four identical Cd_3_(μ^2^-L^naph^)_3_ faces (Fig. 4 and 5). *F*23 is a chiral space group: the absolute configuration could not be determined reliably due to a combination of twinning and low resolution of the diffraction data, but the homochiral arrangement of all metal centres is clear, with the asymmetric unit containing one twelfth of the cage structure (one metal ion, one L^naph^ ligand and one third of an L^mes^ ligand). A view of the entire assembly is in Fig. 4a, and views looking onto the L^mes^-capped face and the Cd_3_(μ^2^-L^naph^)_3_ cyclic helicate face are in Fig. 5. It is clear that the cyclic helicate arrays are stabilised by π-stacking with the naphthyl group of one ligand sandwiched between the coordinated pyrazolyl-pyridine units of the other two. The Cd⋯Cd separations within the helicate faces and the L^mes^-capped faces are 9.97 Å and 11.41 Å respectively.

The crystals that grow in space group *P*2_1_/*n* turned out to be the *C*_3_ symmetric AAAC/CCCA isomer (Fig. 4b). In this case the idealised molecular symmetry is not reflected in the crystal structure, with all 12 Cd(ii) ions being crystallographically inequivalent and hence the asymmetric unit containing an entire molecule. Nonetheless it is clear that one cyclic helicate face has the opposite helical chirality to the other three: this results in one L^mes^ ligand having (non-crystallographic) threefold symmetry and the other three L^mes^ ligands being significantly distorted away from this (Fig. 4b). As in the previous example, the Cd⋯Cd separations around the cyclic helicate faces (9.94–10.41 Å; average 10.16 Å) are considerably shorter than those on the L^mes^-capped faces (average 11.38 Å, with a much wider spread from 10.35 to 12.56 Å). Finally, the crystals that grow in space group *C*2/*c* were the *S*_4_-symmetric AACC isomer (Fig. 4c). The crystallographic symmetry (six independent metal ions, *i.e.* half of the molecule astride a twofold rotation axis) is again lower than the ideal molecular symmetry (with three metal ions, one quarter of the molecule, constituting the asymmetric unit) but the presence of two ‘C’ helicate and two ‘A’ helicate faces is clear. This molecular symmetry requires that all four cyclic helicate faces are structurally equivalent but with all three metal ions within each cyclic helicate being inequivalent; consequently the four L^mes^ ligands are likewise equivalent to one another but have no internal symmetry and those triangular faces have significantly different Cd⋯Cd separations in them. The Cd⋯Cd separations around the helicate faces average 10.06 Å and fall in a very narrow range (10.04–10.09 Å) whereas those on the L^mes^-capped faces are on average longer (11.44 Å) and spread over a wider range (11.04–12.18 Å).

The crystal structures of the three isomers show that the cage molecules enclose large voids whose total volumes can be determined by applying the PLATON-SOLV routine to sets of coordinates from which all solvent and anion entities have been removed and cage apertures artificially blocked. The total volumes of isomers AAAA, AAAC and AACC calculated in this way are 1036, 1121 and 1047 Å^3^ respectively. These minor variations in cavity volumes follow the trend of variations in the volumes of the convex alpha shape defined by the coordinates of the twelve cadmium cations for the isomers: 2872, 2975 and 2951 Å^3^ respectively. Whilst the cage contents and surrounding residues of isomer AAAA cannot be determined from the weak low resolution diffraction data, the contents of the other two cage isomers AAAC and AACC are observed to be a mixture of tetrafluoroborate counterions and solvent molecules (Table S2†). The crystal structures of isomers AAAC and AACC both reveal numerous associations between the cage exterior and interior surfaces with water and acetonitrile solvent residues, mediated by multiple weak C–H⋯O and C–H⋯N hydrogen bonds (Fig. S6–S11†).

We can now understand the ^1^H NMR spectrum (Fig. 2). Although the aromatic region is too complex to provide useful information, the signals from the methyl groups on the L^mes^ ligands (0–3 ppm region) are completely diagnostic as they correlate exactly with the number of Cd environments. Thus for the *T* symmetry isomer we expect one signal arising from all 12 equivalent methyl groups; for the *C*_3_ isomer we expect four different signals, each from three equivalent methyl groups; and for the *S*_4_ isomer we expect three different signals, each from four equivalent methyl groups. A mixture of the three isomers would therefore contain eight signals (sets of 1 + 3 + 4) and this is clear in Fig. 2. The relative intensities of these will depend on the abundance of each isomer in the mixture, which in turn depends not just on simple thermodynamic stability issues but also on the degeneracy of each structure.

**Fig. 6:**
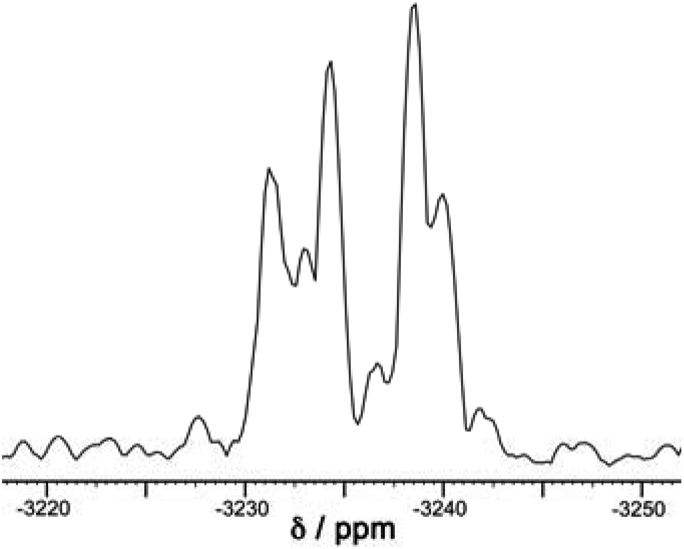
The ^113^Cd NMR spectrum of freshly prepared [Cd_12_(L^naph^)_12_(L^mes^)_4_](BF_4_)_24_ (mixture of isomers) in CD_3_CN. This spectrum is a 1D projection of the F2 dimension taken from the 8.51–6.36 ppm region of the ^1^H dimension of the 600 MHz ^1^H–^113^Cd HMBC spectrum (full spectra available in the ESI†). The broad peaks of overlapping ^113^Cd environments preclude a full deconvolution so we cannot see clear sets of 1, 3 and 4 signals that match the methyl groups in the ^1^H NMR spectrum (Fig. 2), but the spectrum is approximately consistent with the expected number and relative intensities of Cd(ii) environments.

A simple calculation‡‡The *T* isomer requires four cyclic helicates of the same chirality to come together which can only occur in two ways (to give the AAAA/CCCC enantiomers); the arrangement of AAAC or CCCA faces in the *C*_3_ isomer has fourfold degeneracy for each enantiomer, giving 8 possibilities; and the arrangement of AACC faces in the achiral *S*_4_ isomer has sixfold degeneracy with no enantiomer. Thus if there are no steric preferences, and the assembly is dictated simply by chance combination of helical triangular faces with either chirality, the expected 1 : 4 : 6 : 4 : 1 ratio of five stereoisomers (Fig. 3b) would result in *T*, *C*_3_ and *S*_4_ diastereoisomers forming in the ratio 2 : 8 : 6 (= 1 : 4 : 3), *i.e.* 12.5% (*T*) : 50% (*C*_3_) : 37.5% (*S*_4_) of the total population. We also need to factor into this the previous observation that the relative intensities of each methyl signal for the *T*, *C*_3_ and *S*_4_ isomers are, respectively, one signal with intensity 12; four signals of intensity 3; and three signals of intensity 4. We then end up with an expected set of relative intensities as follows: one signal (relative intensity 12) for the *T* isomer; four signals (relative intensity 12 each) for the *C*_3_ isomer; and three signals (relative intensity 12 each) for the *S*_4_ isomer – *i.e.* all eight methyl signals should have the same intensity if assembly of the mixture of cage stereoisomers were purely statistical. suggests that if the distribution of cyclic helicate units amongst the ensemble of the cages were purely statistical, all eight signals from methyl groups would end up with the same intensity. From the ^1^H NMR spectrum this is clearly not the case with the signal for the *T* isomer being relatively weak and the three signals for the *S*_4_ isomer being relatively abundant, with an overall isomer distribution of 5% (*T*), 47.5% (*C*_3_) and 47.5% (*S*_4_) (Fig. 2). This could reflect small differences in thermodynamic stability between the diastereoisomers or could be a kinetic effect associated with different solubilities and crystallisation rates of the three components: we return to this point below. This ratio is repeatable between multiple crystalline samples. The ^113^Cd NMR spectrum (Fig. 6) should follow exactly the same pattern to that found for the methyl groups in the ^1^H NMR spectrum. Although the very similar environments of the Cd(ii) ions will lead to only small differences in chemical shift, we do observe a number of different resonances. Though the signals are not fully resolved, we can identify signals for the different Cd environments with different intensities, partially overlapping but approximately consistent with the ^1^H NMR spectrum.

**Fig. 7:**
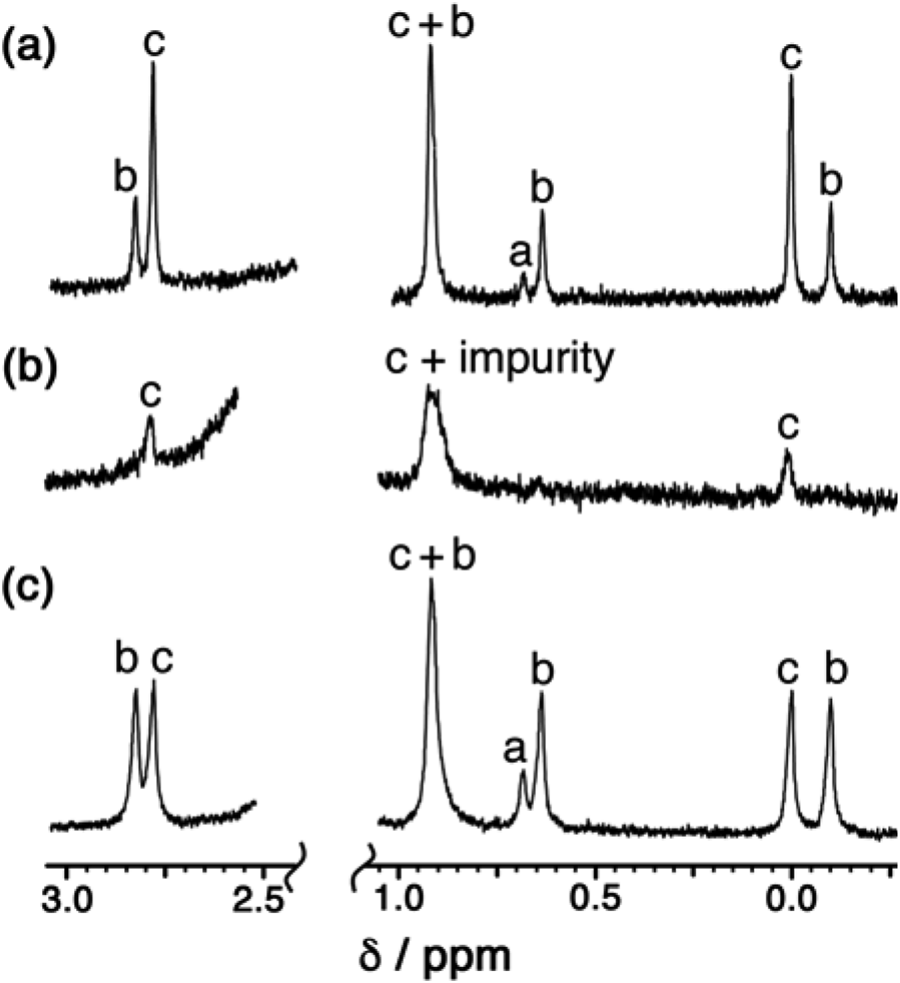
400 MHz ^1^H NMR spectra in CD_3_CN of (a) a recrystallised sample of [Cd_12_(L^naph^)_12_(L^mes^)_4_](BF_4_)_24_ before manual separation of crystal morphologies (this is the same as the spectrum in Fig. 2 except at 400 MHz and is replicated here to aid comparison with the spectra below); (b) manually separated crystals with teeth-like morphology; and (c) manually separated crystals with block morphology. Spectra (b) and (c) of manually selected crystals did not change appreciably over a period of six weeks at room temperature.

Examination of batches of crystals under an optical microscope revealed visibly distinct block-like and teeth-like crystals. Manual selection of the block-like crystals afforded samples which NMR spectroscopy showed to be dominantly the *S*_4_-symmetric stereoisomer; after multiple attempts we were able to obtain a sample of a small number of crystals whose NMR spectrum contained only the three signals (denoted ‘c’ in Fig. 7a) associated with the methyl groups of that isomer (Fig. 7b). Whilst the signal at 0.9 ppm is partly obscured by a solvent impurity, the other two signals at 0.0 and 2.8 ppm are clear; and equally clearly the signals ‘a’ and ‘b’ arising from the *T* and *C*_3_ isomers are absent. Spectra recorded again of this sample after 1 week and then 7 weeks showed no change, implying that the *S*_4_ isomer is kinetically trapped at RT. Manual selection of the teeth-like crystals was less successful at separating the isomers: the NMR spectrum of these samples showed the presence of all three stereoisomers, albeit in a ratio different from that of the bulk sample. The spectrum shown in Fig. 7c shows an excess of the *T* isomer (signal ‘a’), and a deficiency of the *S*_4_ isomer (signals ‘c’), compared to the as-synthesised mixture (Fig. 7a). Again this is kinetically trapped at room temperature, with the non-equilibrium spectrum shown in Fig. 7c not changing significantly after weeks in CD_3_CN solution.

The Cd_12_ cage assembly system is clearly kinetically inert at RT despite the fact that it is based on individually labile Cd(ii) ions. Inverting the chirality of a whole triangular helical panel would require a substantial degree of ligand dissociation as the first step which would present a high activation energy barrier: we have shown before that cages of this general type are very slow (weeks/months) to undergo structural rearrangements^14^ or ligand exchange reactions,^15^ and the same is true of other families self-assembled cages.^16^

Finally, the question arises as to whether the isomer proportions observed in the bulk crystalline sample when it is redissolved (Fig. 2) constitute a thermodynamic minimum, or whether this distribution is a kinetic artefact of the crystallisation process arising from different solubilities of the three isomers. Two experiments suggest that the proportions in the spectrum in Fig. 2 are at, or close to, the thermodynamic minimum. Firstly, we followed the assembly by ^1^H NMR spectroscopy without crystallisation of the components, by combining Cd(BF_4_)_2_ and the two ligands in the correct proportions in CD_3_CN in an NMR tube; the slow appearance of the various methyl signals associated with the Cd_12_ cage between 0 and 3 ppm provided a simple way to follow the assembly process. After 1 h we could see the first signs of the appearance of these signals; and after 2 days the spectrum had stopped evolving (see ESI, Fig. S13†). The spectrum generated by *in situ* assembly was noisy due to solubility limitations of the components so the isomer ratio cannot be determined with high precision, but all three isomers have clearly formed in this experiment in a ratio similar to what was observed in Fig. 2 from the redissolved crystalline sample.

Secondly, we heated a sample of redissolved crystalline material in CD_3_CN to 65 °C for 90 minutes. At this temperature we could see small changes in the proportions of the isomers, with a slight increase in the proportion of the *S*_4_ isomer which then stopped when a new equilibrium was reached after 90 minutes (see ESI, Fig. S14†). Returning the sample to RT resulted in this new slightly changed distribution being kinetically trapped as no further change occurred: the fact that ‘unlocking’ the cage by heating to 65 °C resulted in only a small change in the equilibrium position – which might reasonably be ascribed to the temperature change – implies that the starting solution (prepared from redissolved crystals) was close to equilibrium in the first place.

## Conclusion

In conclusion, we have structurally characterised a unique set of three diastereomeric Cd_12_ coordination cages (*T*, *C*_3_ or *S*_4_ symmetry) based on an overall cuboctahedral array of metal ions with a combination of edge-bridging (ditopic) and face-capping (tritopic) ligands, but displaying different combinations of chirality around the four triangular cyclic helicate faces. In contrast to previous examples of tetrahedral M_4_L_6_ cages where the presence of *T*, *C*_3_ or *S*_4_ symmetry is dictated by the tris-chelate configuration of chelating ligands around the individual metal centres, in this M_12_ cage it arises from the different cyclic helicate arrangements associated with the four triangular Cd_3_(μ^2^-L^naph^)_3_ faces. This source of diastereoisomerism is new to the field of coordination cages and has provided here a set of molecular containers of essentially the same shape and size, but with different cavity chirality. Partial separation of diastereoisomers by manual selection of crystals of different habits was possible. Given the interest in use of chiral molecular containers for various applications based on chiral molecular recognition,^5^ this represents an intriguing and potentially valuable new source of structural diversity. The cages are highly kinetically inert despite being based on labile Cd(ii) ions, with non-equilibrium samples generated by manual selection of crystals with different habits showing no change in their isomer ratio by NMR spectroscopy during weeks at RT.

## Conflicts of interest

There are no conflicts to declare.

## Supplementary Material

SC-011-D0SC04347H-s001

SC-011-D0SC04347H-s002

## References

[cit1] Cook T. R., Stang P. J. (2015). Chem. Rev..

[cit2] Ward M. D., Raithby P. R. (2013). Chem. Soc. Rev..

[cit3] Fujita M. (2000). Struct. Bonding.

[cit4] Metherell A. J., Ward M. D. (2016). Dalton Trans..

[cit5] Chen L.-J., Yang H.-B., Shionoya M. (2017). Chem. Soc. Rev..

[cit6] Caulder D. L., Powers R. E., Parac T. N., Raymond K. N. (1998). Angew. Chem., Int. Ed..

[cit7] Meng W., Clegg J. K., Thoburn J. D., Nitschke J. R. (2011). J. Am. Chem. Soc..

[cit8] Zhang P., Wang X., Xuan W., Peng P., Li Z., Lu R., Wu S., Tian Z., Cu X. (2018). Chem. Commun..

[cit9] Argent S. P., Adams H., Riis-Johannessen T., Jeffery J. C., Harding L. P., Ward M. D. (2006). J. Am. Chem. Soc..

[cit10] This aspect of the chirality of the cuboctahedra was not noticed in the first reported examples (ref. 8) as only a single isomer was isolated

[cit11] Ward M. D., Hunter C. A., Williams N. H. (2018). Acc. Chem. Res..

[cit12] Train J. S., Wragg A. B., Auty A. J., Metherell A. J., Chekulaev D., Taylor C. G. P., Argent S. P., Weinstein J. A., Ward M. D. (2019). Inorg. Chem..

[cit13] Stephenson A., Sykes D., Ward M. D. (2013). Dalton Trans..

[cit14] Ward M. D. (2009). Chem. Commun..

[cit15] Hall B. R., Manck L. E., Tidmarsh I. S., Stephenson A., Taylor B. F., Blaikie E. J., Vander Griend D. A., Ward M. D. (2011). Dalton Trans..

[cit16] Terpin A. J., Ziegler M., Johnson D. W., Raymond K. N. (2001). Angew. Chem., Int. Ed..

