# Supplementary material for: A family of diastereomeric dodecanuclear coordination cages based on inversion of chirality of individual triangular cyclic helicate faces
Source: Chem Sci. 2020 Sep 8;11(37):10167–74. doi: 10.1039/d0sc04347h (PMC8162429; doi:10.1039/d0sc04347h)
Supplement: SC-011-D0SC04347H-s001 [file SC-011-D0SC04347H-s001.pdf]

Electronic Supplementary Material (ESI) for Chemical Science.
This journal is © The Royal Society of Chemistry 2020

A family of diastereomeric dodecanuclear coordination cages based on inversion of chirality of individual triangular cyclic helicate faces

Stephen P. Argent, Fiona C. Jackson, Ho Man Chan, Sam Meyrick, Christopher G. P. Taylor, Tanya K. Ronson, Jonathan P. Rourke and Michael D. Ward

Supporting Information

- **Synthesis**
- **Electrospray Ionisation Mass Spectroscopy**
- **Single Crystal X-ray Diffraction Experiments**
- **Crystal Structure Refinement Experimental Details**
- **Geometric Analysis of the Cages**
- **Additional Nuclear Magnetic Resonance Measurements**

Synthesis

Ligands $\mathbf{L^{mes}}$ and $\mathbf{L^{naph}}$ were prepared according to our previously published methods (main text, refs, 8 and 12 respectively).

$[Cd_{12}(L^{mes})_4(L^{naph})_{12}](BF_4)_{24}$

Ligands $\mathbf{L^{mes}}$ (33.4 mg, 0.0565 mmol) and $\mathbf{L^{naph}}$ (100 mg, 0.226 mmol) were suspended in acetonitrile (30 ml) before addition of $Cd(BF_4)_2$ (69.5 mg, 0.240 mmol, 40 wt% aqueous solution) which caused dissolution of all solids. The solution was stirred overnight at room temperature before the solvent was removed under reduced pressure to give a white powder which was recrystallised from acetonitrile and diisopropyl ether to give **$[Cd_{12}(L^{mes})_4(L^{naph})_{12}](BF_4)_{24}$** (mixture of isomers) as a white crystalline solid which was dried *in vacuo* (yield: 110 mg, 70 %).

Elemental analysis calc. for $Cd_{12}(C_{36}H_{33}N_9)_4(C_{28}H_{22}N_6)_{12}(BF4)_{24}(H_2O)_{40}$: C, 48.73; H, 4.06; N, 12.79. Found: C, 48.75; H, 3.70; N, 12.73 (%).

Electrospray Ionisation Mass Spectroscopy

Measurements were performed on a Bruker Compact Q-TOF mass spectrometer operating in positive mode over the range 200-3000 *m/z* with the ES source operating under the following conditions: dry gas flow 4 L/min; Neb Gas 0.3 bar; Dry Temp 150 °C; Capillary 3599 V; End Plate Offset -500 V; Direct Infusion Flow Rate 3 μL/min. A freshly-made filtered stock solution of **$[Cd_{12}(L^{mes})_4(L^{naph})_{12}](BF_4)_{24}$** in acetonitrile was diluted to a concentration of 1 μM immediately before the measurement.

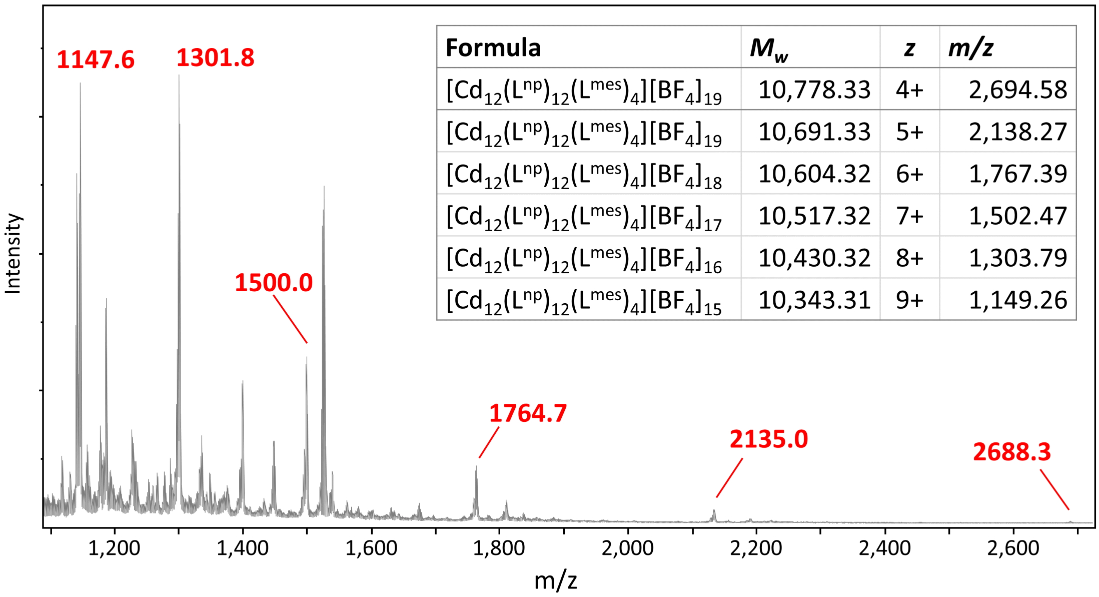

Formula	M_w	z	m/z
$[Cd_{12}(L^{np})_{12}(L^{mes})_4][BF_4]_{19}$	10,778.33	4+	2,694.58
$[Cd_{12}(L^{np})_{12}(L^{mes})_4][BF_4]_{19}$	10,691.33	5+	2,138.27
$[Cd_{12}(L^{np})_{12}(L^{mes})_4][BF_4]_{18}$	10,604.32	6+	1,767.39
$[Cd_{12}(L^{np})_{12}(L^{mes})_4][BF_4]_{17}$	10,517.32	7+	1,502.47
$[Cd_{12}(L^{np})_{12}(L^{mes})_4][BF_4]_{16}$	10,430.32	8+	1,303.79
$[Cd_{12}(L^{np})_{12}(L^{mes})_4][BF_4]_{15}$	10,343.31	9+	1,149.26

Figure S1. ESMS of **$[Cd_{12}(L^{mes})_4(L^{naph})_{12}](BF_4)_{24}$**(mixture of isomers) in acetonitrile. Inset table shows M_w, *z* and *m/z* values calculated for sequential loss of BF_4^- anions from the complex cation. Red labels indicate peaks assigned to the calculated *m/z* values.

Single Crystal X-Ray Diffraction Experimental Details

X-Ray quality single crystals were grown by slow diffusion of diisopropyl ether or diethyl ether into acetonitrile solution of $\mathbf{[Cd_{12}(L^{mes})_4(L^{naph})_{12}](BF_4)_{24}}$. Crystals of three different habits could be identified visually. Many recrystallisations were performed and multiple data sets were collected for each crystal type to try and obtain the best possible data. Single crystals were selected and mounted using Fomblin® (YR-1800 perfluoropolyether oil) on a polymer-tipped MiTeGen MicroMountTM and cooled rapidly to 120 K in a stream of cold N_2 using an Oxford Cryosystems open flow cryostat.[1] Single crystal X-ray diffraction data were collected on an Oxford Diffraction Gemini R (Ruby CCDC detector, fine focus graphite monochromated Cu-Kα radiation source; ω scans) for the AAAA/CCCC isomer or an Oxford Diffraction Supernova diffractometer (Atlas S2 detector, micro focus mirror monochromated Cu-Kα radiation source; ω scans) for AACC and AAAC/ACCC isomers. Cell parameters were refined from the observed positions of all strong reflections and absorption corrections were applied using a Gaussian numerical method with beam profile correction (CrysAlisPro).[2] Structures were solved within Olex2[3] by either direct methods (SHELXS)[4] (AAAA/CCCC isomer) or dual space iterative methods (SHELXT)[5] (AACC and AAAC/ACCC isomers) and refined by full-matrix least-squares on all unique F^2 values with anisotropic displacement parameters (SHELXL).[6] Structures were checked with checkCIF.[7] CCDC 2009535-2009537 contains the supplementary data for these compounds. These data can be obtained free of charge from The Cambridge Crystallographic Data Centre via www.ccdc.cam.ac.uk/data_request/cif.

1. J. Cosier and A. M. Glazer, *J. Appl. Crystallogr.,* 1986, **19**, 105-107.

2. Rigaku Oxford Diffraction, (2018), CrysAlisPro Software system, version 1.171.40.45a, Rigaku Corporation, Oxford, UK.

3. O. V Dolomanov, L. J. Bourhis, R. J. Gildea, J. A. K. Howard, H. Puschmann*, J. Appl. Cryst.,* 2009, **42**, 339–341.

4. G. M. Sheldrick, *Acta Crystallogr. A* 2008, **64**, 112–122.

5. G. M. Sheldrick, *Acta Crystallogr. A* 2015, **71**, 3–8.

6. G. M. Sheldrick, *Acta Crystallogr. C* 2015, **71**, 3–8.

7. “CheckCIF” can be found `t http://checkcif.iucr.org

Table S1. Single crystal X-ray diffraction experimental parameters and refinement information.

	**AAAA-F23_sq**	**AAAC-P21n_sq**	**AACC-C2c_sq**
Chemical formula	$C_{640}H_{644}B_{24}Cd_{12}F_{96}N_{188}O_4$	$C_{560}H_{556}B_{24}Cd_{12}F_{96}N_{148}O_{20}$	$C_{504}H_{472}B_{24}Cd_{12}F_{96}N_{120}O_{20}$
M_r	14465.63	13111.73	11962.23
Crystal system, space group	Cubic, *F*23	Monoclinic, $P2_1/n$	Monoclinic, *C*2/*c*
Z	4	4	4
a, *b*, *c* (Å)	42.9589(6), 42.9589(6), 42.9589(6)	26.5845(8), 53.3766(10), 44.0442(8)	89.68(3), 24.7801(10), 51.463(19)
α, β, γ (°)	90, 90, 90	90, 94.842(2), 90	90, 147.82(9), 90
V (Å^3)	79279 (3)	62275 (2)	60909 (87)
Radiation	Cu $K\alpha$	Cu $K\alpha$	Cu $K\alpha$
μ (mm^{-1})	3.22	4.04	4.07
Temperature	150 K	150 K	150 K
Crystal size (mm)	0.19 × 0.16 × 0.08	0.55 × 0.18 × 0.11	0.22 × 0.16 × 0.08
Diffractometer and detector	Gemini Xcalibur, Ruby	SuperNova, AtlasS2	SuperNova, AtlasS2
T_{min}, T_{max}	0.490, 0.752	0.198, 1.000	0.571, 0.985
No. of measured, independent and observed [$I > 2\sigma(I)$] reflections	22267, 2076, 1801	171300, 48974, 30862	56697, 20807, 13213
R_{int}	0.052	0.152	0.054
θ_{max} (°)	30.9	44.5	42.1
$(\sin \theta/\lambda)_{max}$ (Å^{-1})	0.333	0.455	0.435
$R[F^2 > 2\sigma(F^2)]$, $wR(F^2)$, S	0.110, 0.329, 1.79	0.151, 0.418, 1.43	0.129, 0.416, 1.57
No. of reflections	2076	48974	20807
No. of parameters	457	5868	2870
No. of restraints	916	36632	11142
$\Delta\rangle_{max}$, $\Delta\rangle_{min}$ (e Å^{-3})	0.88, -0.35	1.26, -1.07	1.42, -0.58
CCDC Deposit Number	2009535	2009536	2009537

Crystal Structure Refinement Experimental Details

AAAA-F23_sq

Refined as a 4-component inversion twin with twin law {0 -1 0 -1 0 0 0 0 -1} and three batch scale factors refining as 0.42(8), 0.42(8) and 0.07(8). Alternative twin laws were tested, all giving the same improvements in refinement and R-values. No Flack parameter is calculated for the refinement of the twinned structure. The X-ray diffraction intensity dropped off at high angle with a diffraction limit of 1.6 Å. As a result of the low-resolution diffraction limit the structure refinement has a low data to parameter ratio of 4.54:1. Extensive use of restraints on geometric and displacement parameters is used to aid the refinement.

An initial solution using direct methods (ShelXS) gave a plausible position for a cadmium atom and one pyrazolyl-pyridine moiety. The remaining ligand atoms were generated from idealised fragment libraries before being refined under the influence of extensive geometric restraints generated by Grade Web Server (DFIX, DANG, FLAT). Anti-bumping restraints have been applied to the nitrogen-cadmium distances for N31B and N21A (DFIX -2.3) and a target distance of 2.45 Å has been applied to the Cd1-N11A distance (DFIX). Geometric similarity restraints have been applied to the 1,3 and 1,4 distances around the trismethylene-mesityl moiety to maintain three-fold symmetrical geometry (SADI).

The anisotropic displacement parameters of the carbon and nitrogen atoms are restrained to be similar (SIMU) and to have a rigid bond approximation (RIGU). The anisotropic displacement parameters of the carbon atoms of the mesityl moiety are restrained to have more isotropic character (ISOR). The anisotropic displacement parameters are universally very large likely as a result of the poor crystallinity of the macromolecule species surrounded by large regions of diffuse solvent and anions. Water residue O1W is refined with an isotropic displacement parameter.

All hydrogen atoms were geometrically placed and refined with a riding model. Hydrogen atoms of water residue O1W were not observed in the electron density map; they are not included in the model, however, they are included in the unit cell contents and all values derived from it.

PLATON SQUEEZE was applied to the data to remove scattering contributions from several disordered anion and solvent residues which could not be modelled as discrete sites: 11121 electrons per unit cell were consistent with two tetrafluoroborate anions and 6.67 acetonitrile solvent molecules per asymmetric unit. These molecules have been included in the chemical formula and in all values derived from it. SQUEEZE also yielded a set of solvent-free diffraction intensities for use in the final cycles of refinement.

AACC-C2c_sq

The X-ray diffraction intensity of this large supramolecular assembly featuring large regions of diffuse anions and solvent residues dropped off rapidly at higher angles. The data used in the refinement was truncated to an upper resolution of 1.15 Å. As a result of the low-resolution diffraction limit, the refinement has a data to parameter ratio of 7.25:1. Extensive use of restraints on geometric and displacement parameters aided the refinement. Only atoms of the central metallo-cage residue were refined with isotropic displacement parameters.

The 1,2 and 1,3 distances in the pyrazolylpyridine moieties, naphthyl moieties and tetrafluoroborate anions were restrained to be similar (SAME). The 1,2 bond distances of the methylene moieties to pyrazolyl and naphthyl moieties were respectively restrained to be similar (SADI). The geometries of several pyridine, pyrazole and naphthyl ring systems were restrained to be planar (FLAT). All of the 1,2 bond distances in the pyrazolylpyridine moieties were restrained to have suitable target values taken from averages of equivalent moieties in the CSD (DFIX).

The anisotropic displacement parameters of the carbon, nitrogen, boron and fluorine atoms were restrained to be similar (SIMU) and to have a rigid bond approximation (RIGU). The tetrafluoroborate and water residues are refined with isotropic displacement parameters. The isotropic displacement parameters of all water residues were fixed at a value of 0.2. The isotropic displacement parameters of tetrafluoroborate residues B11, B31, B41, B71 and B81 were fixed at a value of 0.25. The occupancies of tetrafluoroborate and water residues were allowed to refine, using a common free variable in the case of each tetrafluoroborate residue. Tetrafluoroborate residues B11/B81 and water residues O1W/O1X are disordered pairs respectively; their refined occupancies are constrained to sum to unity.

Some small residual electron density peaks close to water residues indicate that they might be substitutionally disordered with acetonitrile solvent residues (crystallisation solvent). No sensible model could be developed for any such disorder sites. All hydrogen atoms were geometrically placed and refined with a riding model. Hydrogen atoms of water residues were not observed in the electron density map; they are not included in the model, however, they are included in the unit cell contents and all values derived from it. Some short H...H intramolecular contacts are observed between mesitylmethyl and methylene hydrogen atoms. These are caused use of idealised methyl hydrogen positions rather than positions derived from refinement of the torsion angle for which the data quality was insufficient. The clashing methyl hydrogen atoms are retained in the model rather than omitted to give the model more realistic cumulative scattering strength.

PLATON SQUEEZE was applied to the data to remove scattering contributions from several disordered anion and solvent residues which could not be modelled as discrete sites: 4335 electrons per P1 unit cell and a volume of 19877 Å^3 were equated with seven tetrafluoroborate anions, twelve acetonitrile solvent molecules and six water molecules per asymmetric unit. These molecules have been included in the chemical formula and in all values derived from it. SQUEEZE also yielded a set of solvent-free diffraction intensities for use in the final cycles of refinement.

AAAC-P21n_sq

The weakly diffracting crystals of this large supramolecular metallo-cage features large regions of poorly crystalline anion, solvent and water molecules and as a result, had a low-resolution diffraction limit. The data used for the refinement were truncated to a resolution of 1.1 Å resulting in a low data to parameter ratio of 8.3:1. Extensive use of restraints on geometric and displacement parameters aided the refinement. Only atoms of the central metallo-cage residue were refined with isotropic displacement parameters.

The 1,2 and 1,3 distances in the pyrazolylpyridine moieties, naphthyl moieties, diethyl ether solvent residues and tetrafluoroborate anions were restrained to be similar (SAME). The 1,2 bond distances of the methylene moieties to pyrazolyl and naphthyl moieties were respectively

restrained to be target values (DFIX). The geometries of all pyridine, pyrazole and naphthyl ring systems were restrained to be planar (FLAT). All acetonitrile solvent residues were refined as rigid bodies with idealised coordinates taken from a fragment library (Guzei, I. A. *J. Appl. Crystallogr.* 2014, **47**, 806-809). Hydrogen atoms were omitted for water residues and some acetonitrile residues where the hydrogen torsion angle did not converge.

The atoms of the metallo-cage complex were refined with anisotropic displacement parameters; rigid bond and similarity restraints were applied to the parameters of the carbon and nitrogen atoms (RIGU, SIMU). All other atoms in the structure (solvent residues and tetrafluorobrate anions) were refined with isotropic displacement parameters. To reduce the parameter burden on the refinement a common value for the isotropic displacement parameters was refined for the non-hydrogen atoms of each acetonitrile residue and tetrafluoroborate anion. The occupancies of tetrafluoroborate anions B31, B111, B171 and B191 were refined, whilst the occupancy of B101 was fixed at a half. The occupancies of acetonitrile residues Q, Y and X were refined. The isotropic displacement parameters of water residues 1-9 and 21-29 were fixed at a value of 0.25. The occupancies of water residues 3, 6, 7, 27, 28, and 29 were refined.

Some small residual electron density peaks close to water residues indicate that they might be substitutionally disordered with acetonitrile solvent residues (crystallisation solvent). No sensible model could be developed for any such disorder sites.

All hydrogen atoms were geometrically placed and refined with a riding model. Hydrogen atoms of water residues were not observed in the electron density map; they are not included in the model, however, they are included in the unit cell contents and all values derived from it. PLATON SQUEEZE was applied to the data to remove scattering contributions from several disordered anion and solvent residues which could not be modelled as discrete sites: 3675 electrons per P1 unit cell and a volume of 14,249 $Å^3$ were equated with six tetrafluoroborate anions and 30 acetonitrile solvent molecules per asymmetric unit. These molecules have been included in the chemical formula and in all values derived from it. SQUEEZE also yielded a set of solvent-free diffraction intensities for use in the final cycles of refinement.

Geometric analysis of the cages

Cavity volumes inside the crystallised cage complexes were determined using the SOLV routine in PLATON. Input files were prepared from the final refined CIFs by manually removing solvent and anion residues from inside the cage cavities and blocking the cage apertures with artificial dummy-atoms (sulphur or iodine).

The geometric volume enclosed the cuboctahedral array of twelve cadmium atoms in each crystal structure was determined using the alphaShape algorithm in the software MATLAB online R2020a. The coordinates of the metal cations for a complete cage moiety were exported from Olex2 v1.3 as an XYZ file before being defined as a 12x3 matrix *A* in MATLAB:

```
A = [11.2466   24.6533   18.2880; 18.2880   32.7261    3.1738;
18.2880   31.7123   18.3056; 3.1738   24.6709   10.2328; 10.2328
24.6533    3.1914; 18.3056   24.6709   11.2466; 3.1914   31.7123
3.1738; 3.1914   32.7261   18.3056; 3.1738   39.7675   11.2466;
10.2328   39.7851   18.2880; 18.3056   39.7675   10.2328; 11.2466
39.7851    3.1914]
```

The coordinates were defined as an alpha shape:

```
CUBO = alphaShape(A)
```

An alpha spectrum was determined to find the critical alpha radius (largest value listed in the alpha spectrum) necessary to generate a convex alpha shape:

```
alphaSpectrum(CUBO)

CUBO.Alpha = 13
```

The cuboctahedron was plotted to ensure all vertices were appropriately connected and the volume determined:

```
plot(CUBO)

volume(CUBO)
```

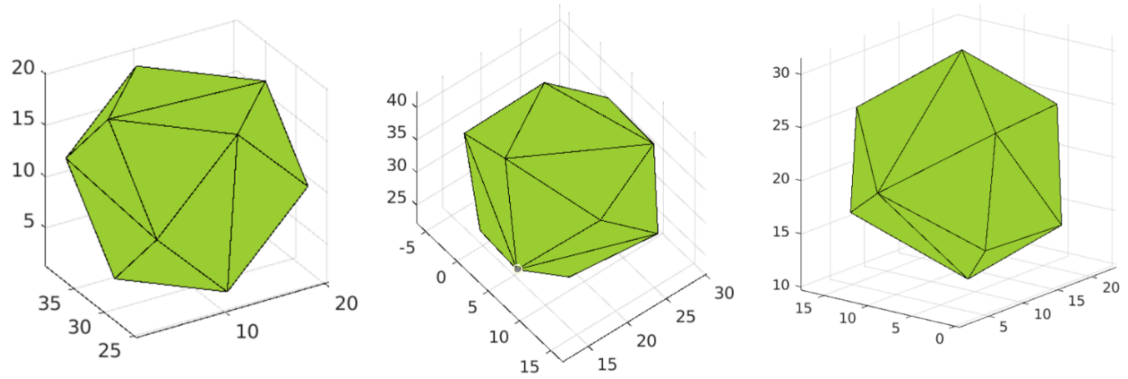

AAAA AAAC AACC

Figure S2. Plots of alpha shapes for cuboctahedral arrays defined by Cd cation positions from crystal structures of diasteromers AAAA, AAAC and AACC using the 'alphaShape' function in MATLAB.

Table S2. Crystallographically determined volumes and contents of coordination cages.

Cage	Total internal cage volume (PLATON) (Å^3)	Volume accessible to atom centres (PLATON) (Å^3)	{Cd_{12}} Polyhedron alpha shape volume (MATLAB) (Å^3)	Cage contents observed in crystal structure
**AAAA**	1036	369	2872	Diffuse - SQUEEZED
**AAAC**	1121	420	2975	{$(BF_4)_6(CH_3CN)_2(H_2O)_4Et_2O$}
**AACC**	1047	409	2951	{$(BF_4)_2(H_2O)_4$}

To aid visual inspection of the structures and the determination of the molecular point group of each cage, cardboard nets of cuboctahedra decorated with the principal symmetry features of each cage were printed, cut out and assembled using UHU adhesive. The nets are included here as an aid to visualisation of the of the differing symmetries of the diastereomeric cages.

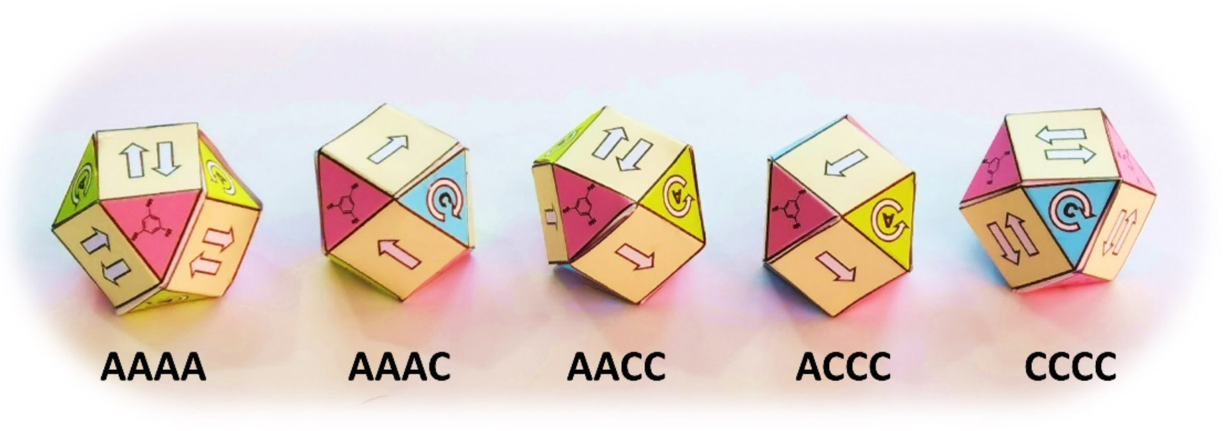

Figure S3. 3D Models of depicting the relative helicities of the faces for the five stereoisomers of **[$Cd_{12}(L^{mes})_4(L^{naph})_{12}](BF_4)_{24}$** (two enantiomeric pairs for the two diastereoisomers AAAA/CCCC and AAAC/ACCC, and the achiral diastereoisomer AACC).

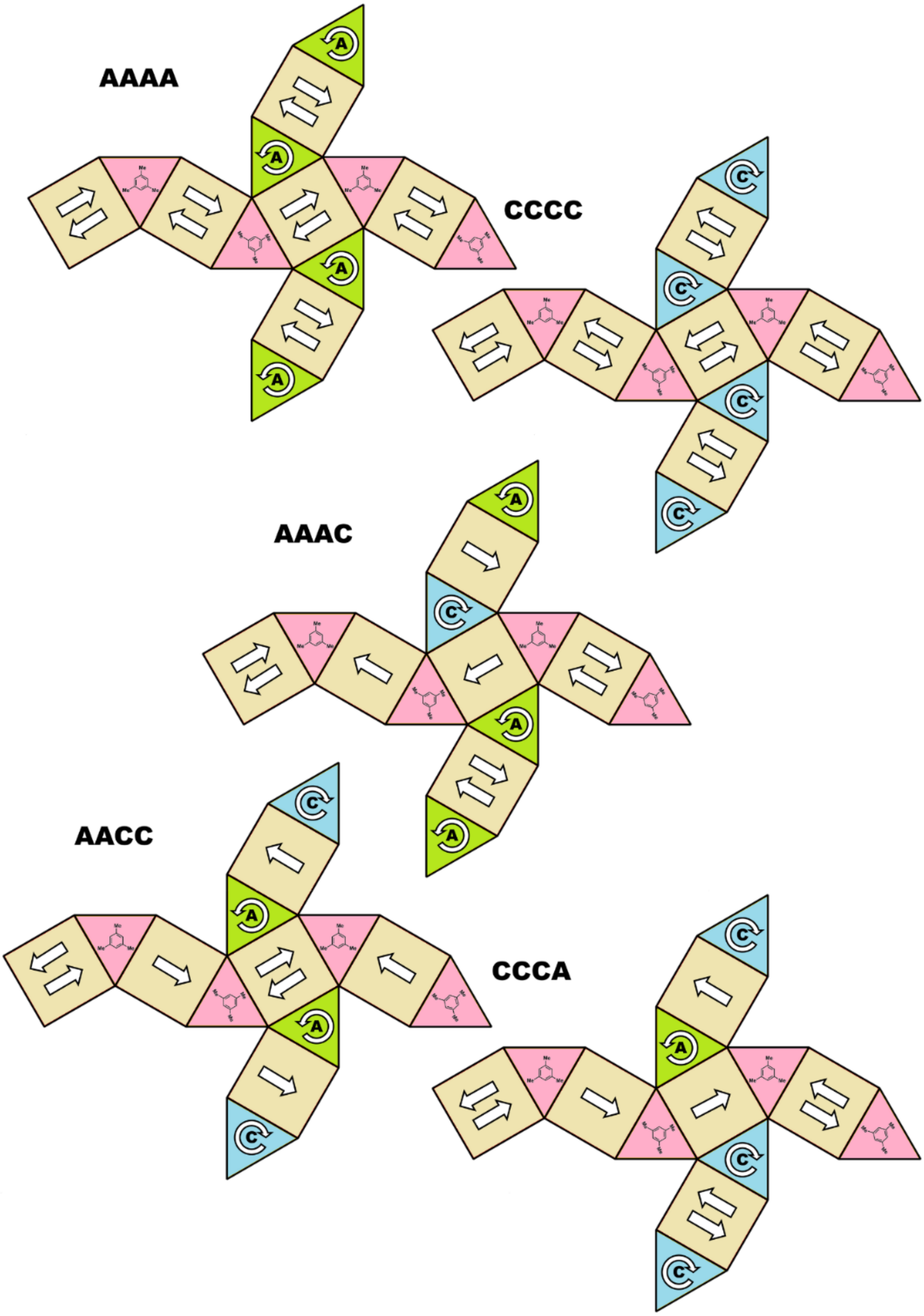

Figure S4. Printable nets depicting the relative symmetry elements of the five stereoisomers of **$[Cd_{12}(L^{naph})_{12}(L^{mes})_4](BF_4)_{24}$.** Curly arrows denote the helicity of the $\{Cd_3(L^{naph})_3\}$ triangular helicate faces. Arrows on square faces denote the helicity of adjacent triangular helical faces. Tabs should be left adjacent to all triangular edges when cutting out the shapes. Score folds with scissors (get a grown-up or professor to help you) and stick together using a fast contact adhesive in a well-ventilated area.

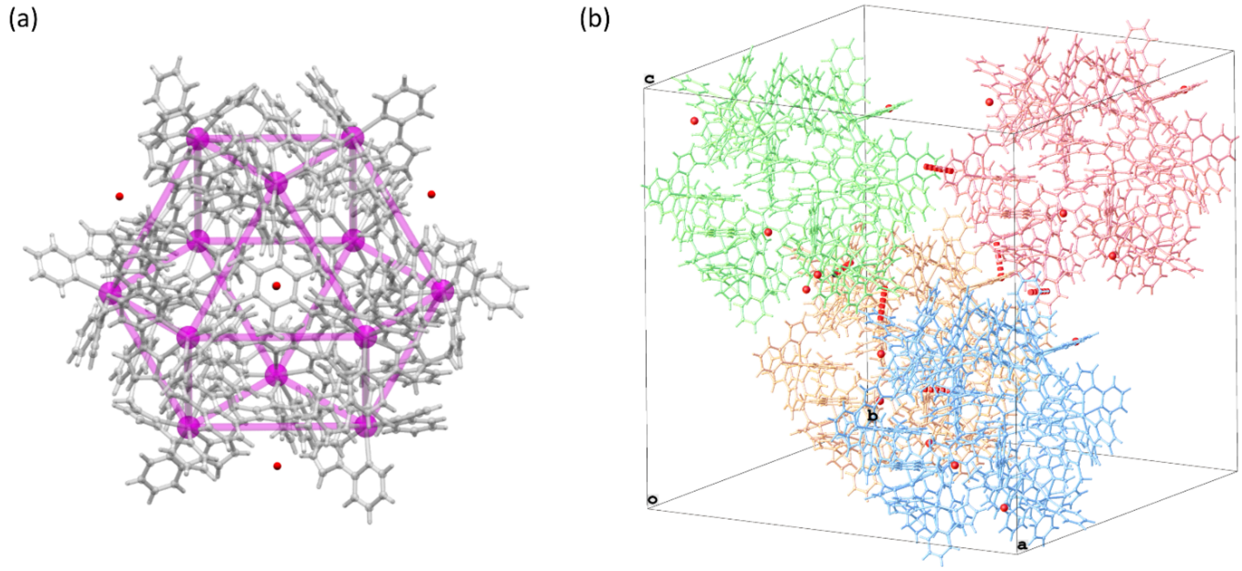

Figure S5. Views of the crystal structure of **$[Cd_{12}(L^{naph})_{12}(L^{mes})_4](BF_4)_{24}$ (AAAA-diastereomer)**: (a) Single complex cage cation highlighting cuboctahedral cadmium cage topology (purple spheres and lines) and four water residues located outside the cage adjacent to the mesitylene rings (red); (b) Packing diagram showing the arrangement of four complex cage cations in the unit cell including water residues (red spheres) and intermolecular π–π interactions between pyridyl rings of adjacent cages (ring centroid-centroid distance 3.79(4) Å, centroid-mean plane distance 3.69(5) Å) as bold red dashed lines.

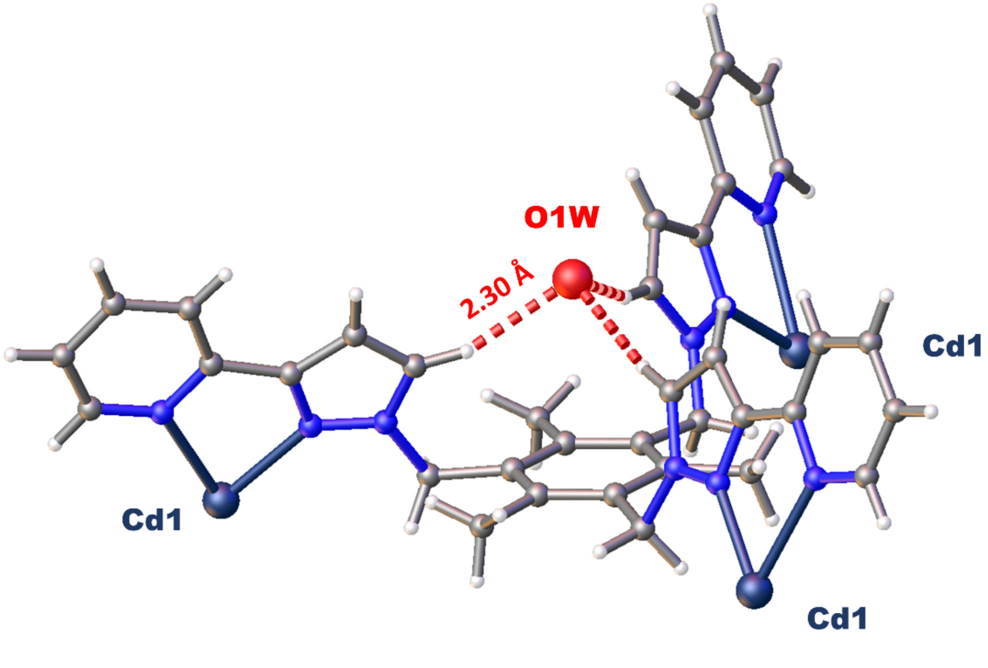

Figure S6. View of the environment of coordinated ligand L^{mes} from the crystal structure of **$[Cd_{12}(L^{naph})_{12}(L^{mes})_4](BF_4)_{24}$ (AAAA-diastereomer)** including short C-H···O contacts between the pyrazolyl C-H groups and water residue O1W (red dashed lines).

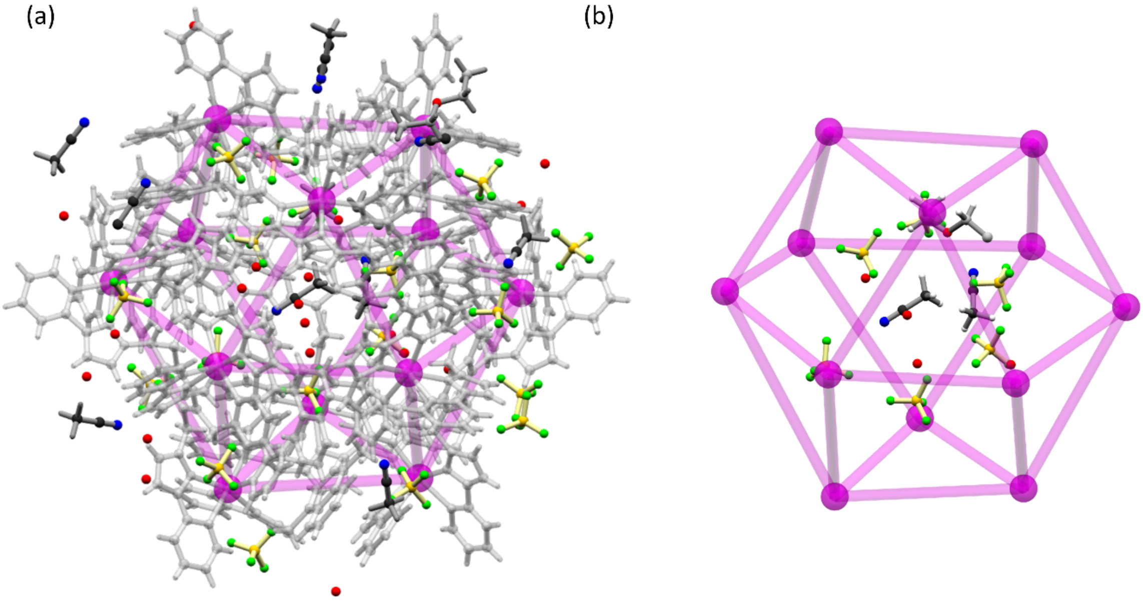

Figure S7. Views of the crystal structure of **$[Cd_{12}(L^{naph})_{12}(L^{mes})_4](BF_4)_{24}$ (AAAC-diastereomer)**: (a) Complex cage cation with internal cage contents and immediate shell of surrounding anions and solvent residues; (b) view of the cage contents within the cuboctahedral shell.

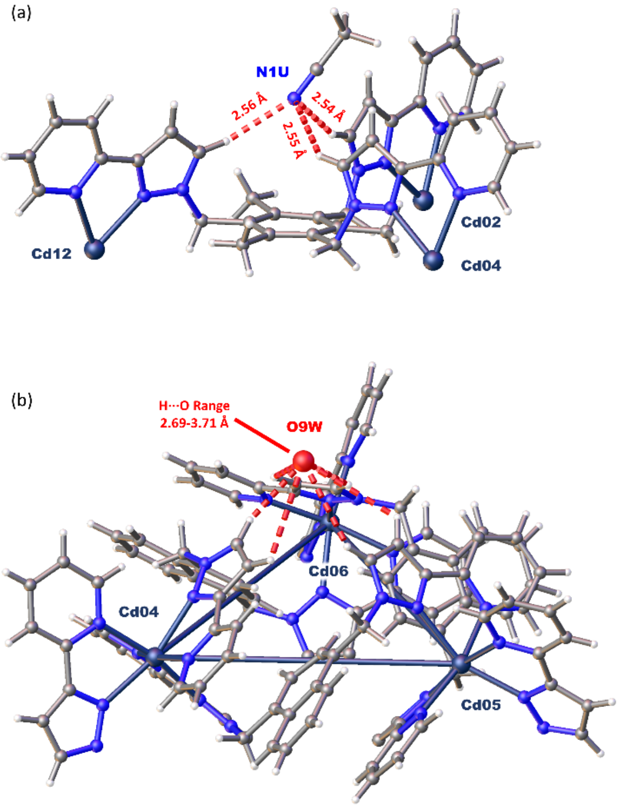

Figure S8. View of the environments of (a) coordinated ligand L^{mes} and (b) $\{Cd_3(L^{naph})_3\}$ cyclic helicate sub-unit from the crystal structure of **$[Cd_{12}(L^{naph})_{12}(L^{mes})_4](BF_4)_{24}$ (AAAC-diastereomer)** including short contacts between the pyrazolyl C-H groups and water/acetonitrile solvent residues (red dashed lines).

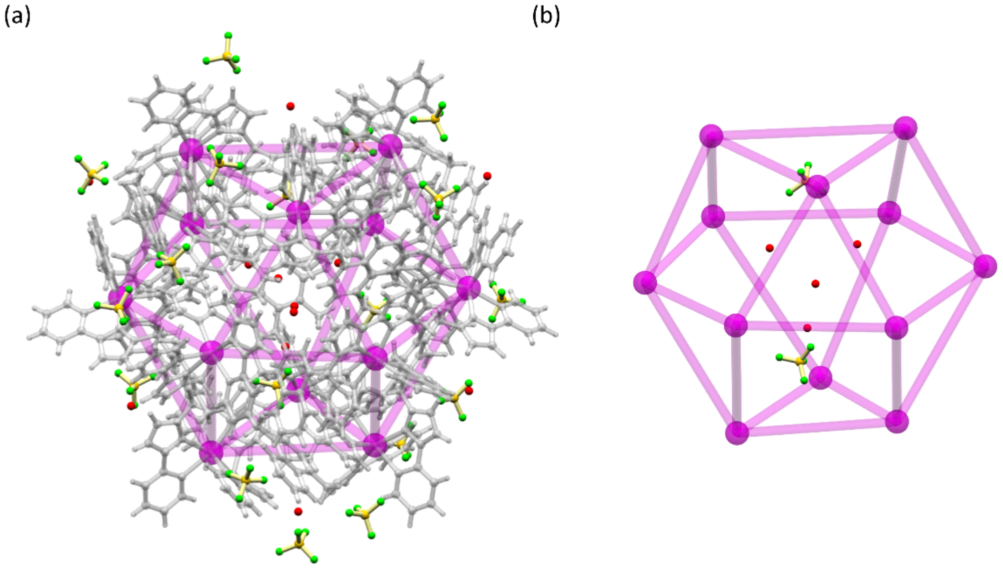

Figure S9. Views of the crystal structure of **$[Cd_{12}(L^{naph})_{12}(L^{mes})_4](BF_4)_{24}$ (AACC-diastereomer)**: (a) Complex cage cation with internal cage contents and immediate shell of surrounding anions and solvent residues; (b) view of the cage contents within the cuboctahedral shell.

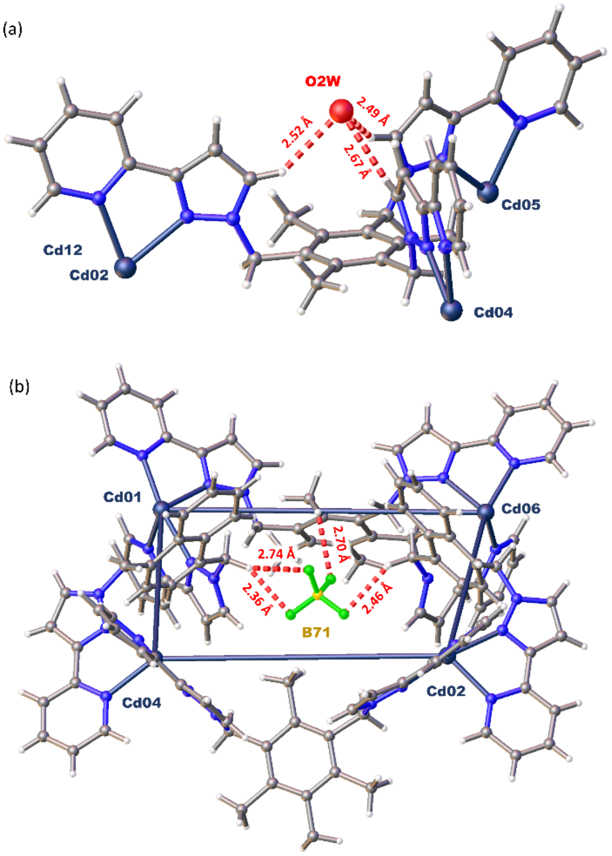

Figure S10. View of the environments of (a) coordinated ligand L^{mes} and (b) $\{Cd_4(L^{mes})_2(L^{naph})_4\}$ square sub-unit from the crystal structure of **$[Cd_{12}(L^{naph})_{12}(L^{mes})_4](BF_4)_{24}$ (AACC-diastereomer)** including short contacts between the pyrazolyl C-H groups and water solvent residue and the methylene C-H groups and tetrafluoroborate fluorine atoms (red dashed lines).

NMR Spectroscopy

High field ^{1}H and ^{113}Cd NMR spectra were measured on a Bruker Avance III 600 MHz spectrometer. ^{1}H-^{113}Cd correlation spectra were recorded using a variant of the HMBC pulse sequence and the ^{113}Cd chemical shifts reported are taken from these spectra (referenced to external 0.1 M [Cd(ClO)$_4$)$_2$]). The spectra of **[$Cd_{12}(L^{naph})_{12}(L^{mes})_4](BF_4)_{24}$** (mixture of isomers) were measured in CD_3CN solution. The ^{113}Cd spectrum is a 1D Projection of the F2 dimension taken from the 8.51-6.36 ppm region of the 1H dimension of the 600 MHz ^{1}H-^{113}Cd HMBC spectrum. The full spectrum is dominated by cross peaks resulting from the large quantity of residual solvent. The broad peaks of overlapping ^{113}Cd environments preclude a full deconvolution, however, the spectrum is consistent with the expected presence of eight environments (see main text).

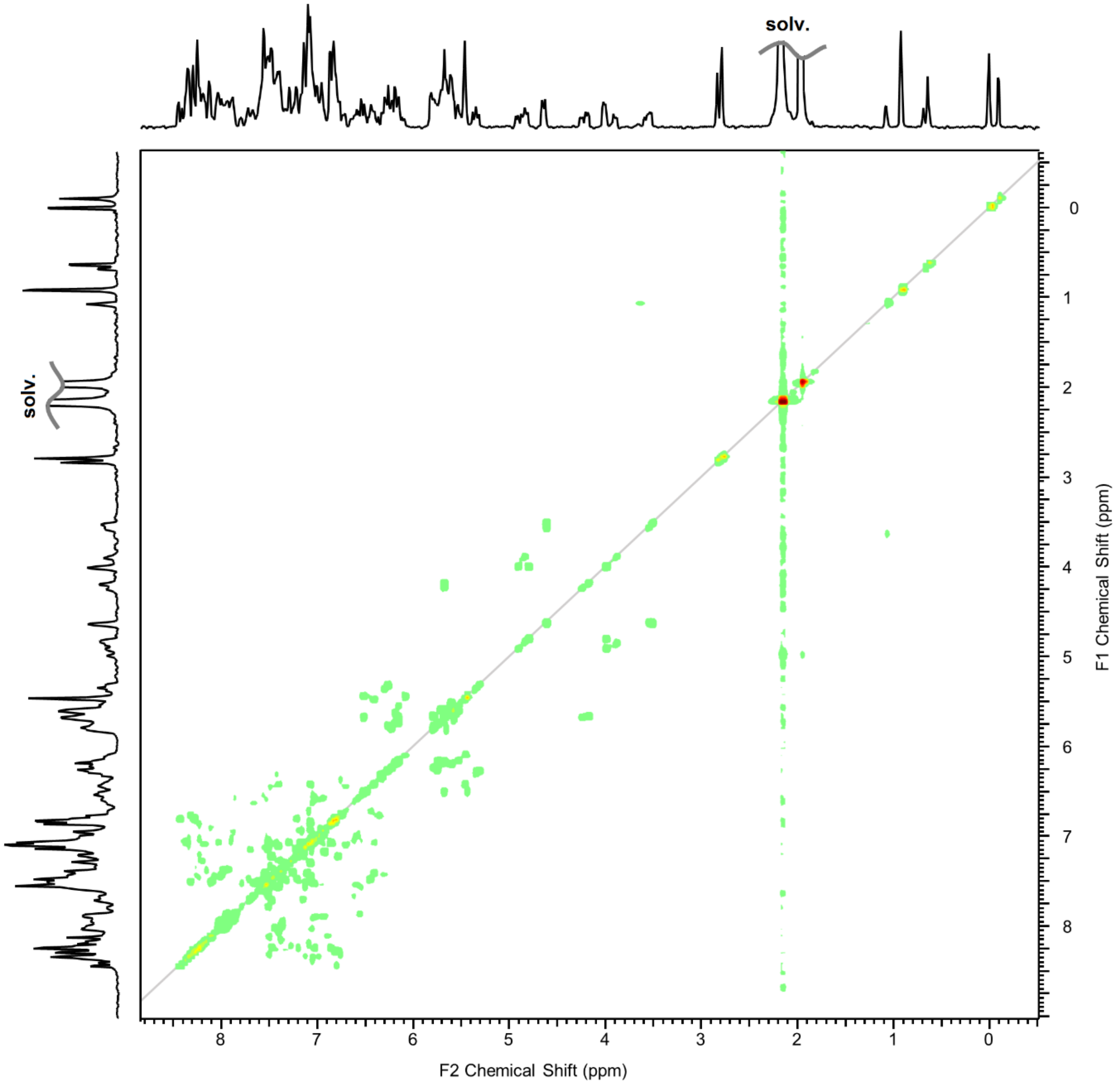

Figure S11. ^{1}H-^{1}H COSY NMR (600 MHz, CD_3CN) of **[$Cd_{12}(L^{naph})_{12}(L^{mes})_4](BF_4)_{24}$** (mixture of isomers).

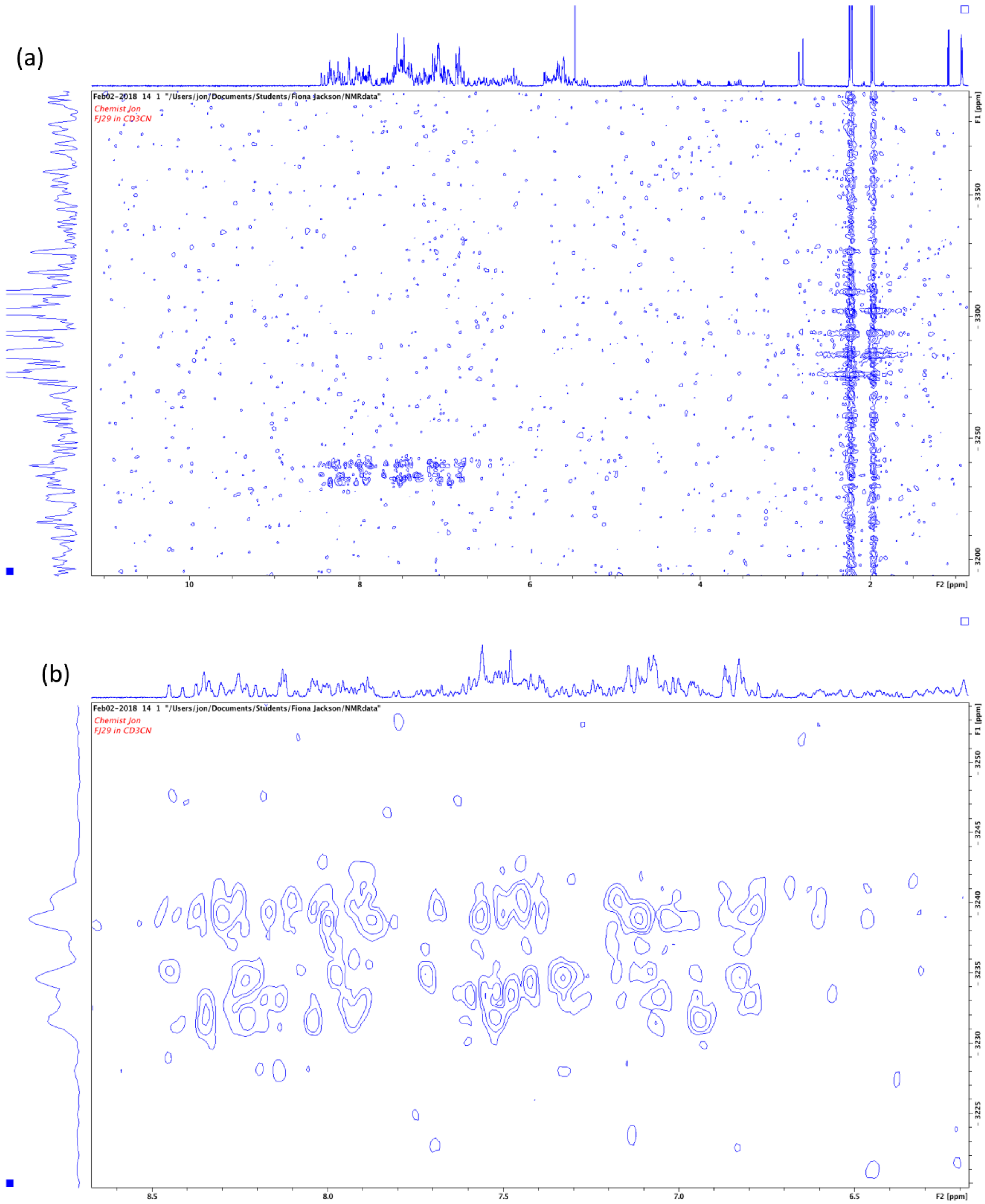

Figure S12. ^{1}H-^{113}Cd HMBC NMR (CD_3CN, 600 MHz) of **$[Cd_{12}(L^{naph})_{12}(L^{mes})_4](BF_4)_{24}$** (mixture of isomers) for (a) full area and (b) cropped area featuring cross peaks between ligand aromatic proton peaks and cage complexed cadmium cations. In both plots the vertical axis curve is a 1D projection of the F2 dimension shown in the 2D plot area.

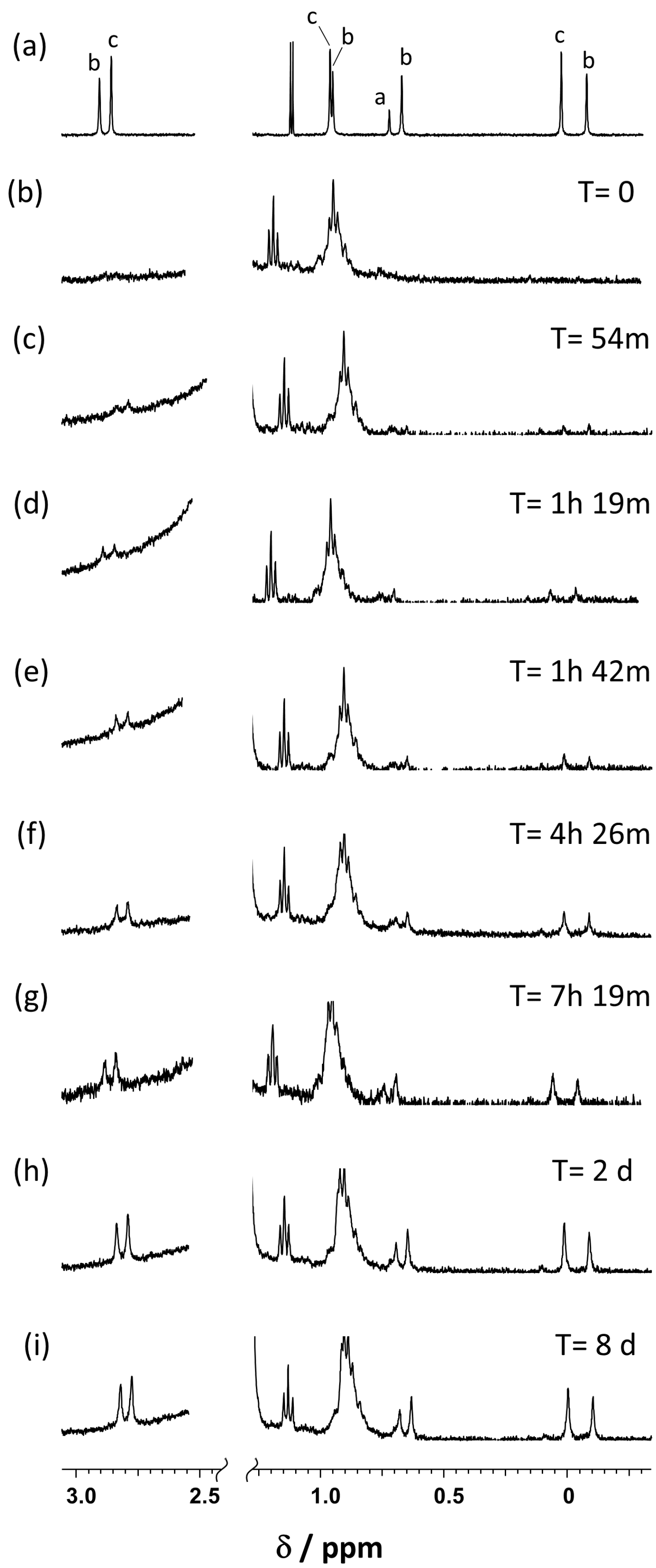

Figure S13. (a) 600 MHz ^{1}H NMR spectra in CD_3CN of a recrystallised sample of $[Cd_{12}(L^{naph})_{12}(L^{mes})_4](BF_4)_{24}$ (same as Fig. 2, main text); (b) - (i) 400 MHz ^{1}H NMR spectra in CD_3CN at 298 K of a mixture of L^{naph} L^{mes}, and $Cd(BF_4)_2$ in a 3:1:3 ratio, showing formation of the cuboctahedral cage over a period of 8 days.

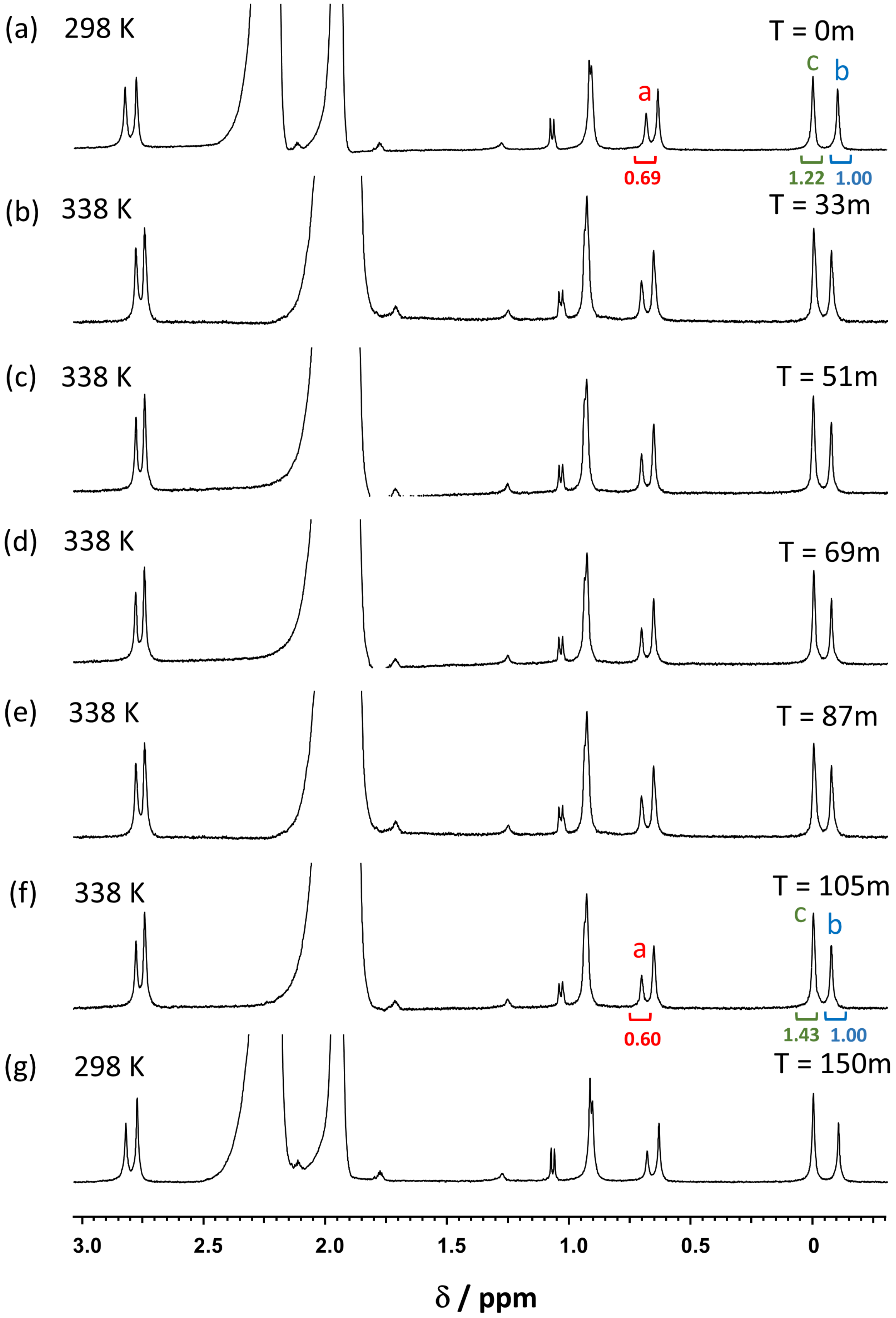

Figure S14. 400 MHz ^{1}H NMR spectra in CD_3CN of a recrystallised sample of $[Cd_{12}(L^{naph})_{12}(L^{mes})_4](BF_4)_{24}$ (a) at 298 K before heating, (b)-(f) at 338 K during heating and (g) at 298 K after heating. Integrals at the start [spectrum (a)], and at the end of the heating period [spectrum (g)], are included: for meaning of the labels 'a', 'b' and 'c' see main text.
